# Candidate Luminal B Breast Cancer Genes Identified by Genome, Gene Expression and DNA Methylation Profiling

**DOI:** 10.1371/journal.pone.0081843

**Published:** 2014-01-09

**Authors:** Stéphanie Cornen, Arnaud Guille, José Adélaïde, Lynda Addou-Klouche, Pascal Finetti, Marie-Rose Saade, Marwa Manai, Nadine Carbuccia, Ismahane Bekhouche, Anne Letessier, Stéphane Raynaud, Emmanuelle Charafe-Jauffret, Jocelyne Jacquemier, Salvatore Spicuglia, Hugues de The, Patrice Viens, François Bertucci, Daniel Birnbaum, Max Chaffanet

**Affiliations:** 1 Marseille Cancer Research Center, UMR1068 Inserm, Institut Paoli-Calmettes (IPC), Department of Molecular Oncology, Equipe labellisée Ligue Contre le Cancer, Marseille, France; 2 Biotoxicology Laboratory, Djillali Liabes University, Sidi-Bel-Abbès, Algeria; 3 Biochemistry and Molecular Biology Unit, Biology Dpt, Faculty of Sciences El Manar, Tunis, Tunisia; 4 Institut Paoli-Calmettes (IPC), Department of Biopathology, Marseille, France; 5 Aix-Marseille Université, Marseille, France; 6 Inserm U1090, TAGC, Marseille, France; 7 UMR 944/7212 INSERM/CNRS/Université Paris 7, Hôpital St Louis, Paris, France; 8 Institut Paoli-Calmettes (IPC), Department of Medical Oncology, Marseille, France; Baylor College of Medicine, United States of America

## Abstract

Breast cancers (BCs) of the luminal B subtype are estrogen receptor-positive (ER+), highly proliferative, resistant to standard therapies and have a poor prognosis. To better understand this subtype we compared DNA copy number aberrations (CNAs), DNA promoter methylation, gene expression profiles, and somatic mutations in nine selected genes, in 32 luminal B tumors with those observed in 156 BCs of the other molecular subtypes. Frequent CNAs included 8p11-p12 and 11q13.1-q13.2 amplifications, 7q11.22-q34, 8q21.12-q24.23, 12p12.3-p13.1, 12q13.11-q24.11, 14q21.1-q23.1, 17q11.1-q25.1, 20q11.23-q13.33 gains and 6q14.1-q24.2, 9p21.3-p24,3, 9q21.2, 18p11.31-p11.32 losses. A total of 237 and 101 luminal B-specific candidate oncogenes and tumor suppressor genes (TSGs) presented a deregulated expression in relation with their CNAs, including 11 genes previously reported associated with endocrine resistance. Interestingly, 88% of the potential TSGs are located within chromosome arm 6q, and seven candidate oncogenes are potential therapeutic targets. A total of 100 candidate oncogenes were validated in a public series of 5,765 BCs and the overexpression of 67 of these was associated with poor survival in luminal tumors. Twenty-four genes presented a deregulated expression in relation with a high DNA methylation level. *FOXO3, PIK3CA* and *TP53* were the most frequent mutated genes among the nine tested. In a meta-analysis of next-generation sequencing data in 875 BCs, *KCNB2* mutations were associated with luminal B cases while candidate TSGs *MDN1* (6q15) and *UTRN* (6q24), were mutated in this subtype. In conclusion, we have reported luminal B candidate genes that may play a role in the development and/or hormone resistance of this aggressive subtype.

## Introduction

Breast cancer (BC) is a complex and heterogeneous disease whose therapeutic approach must be refined in view of recent studies allowing better classification and/or prognosis assessment [1]. DNA microarray-based expression profiling has identified clinically and biologically relevant intrinsic molecular subtypes (luminal A, luminal B, ERBB2, basal, and normal-like) [2]–[4] and prognostic and/or predictive gene expression signatures [5]. Genomic studies have also suggested a prognostic impact of genomic data [6]–[8]. Combining expression and genomic data allowed the identification of candidate BC genes [6], [9]–[19]. The status of DNA methylation may also contribute to improve BC molecular classification [20]–[24]. The identification of new fusion genes by RNA-seq approaches [25], [26] and of driver mutations in cancer genes in various molecular and clinical BC entities [27]–[31] will also help design targeted treatments.

Luminal B BCs have a poor prognosis [32]. Although they express hormone receptors, their metastatic risk and resistance to hormone therapy and to conventional chemotherapy demand to develop appropriate therapies. Some proteins (e.g. CITED2, NCOR2) or molecular networks associated with *BCAR* (breast cancer anti-estrogen resistance) genes are involved in the resistance to anti-estrogen therapy and in the progression of these cancers [32]–[35]. Luminal B cancers exhibit various mutated genes, *TP53* and *PIK3CA* being the most frequent (29% each) [28].

To further define molecular alterations associated with the luminal B subtype we studied DNA copy number aberrations (CNAs), DNA promoter methylation alterations (DPMAs), gene expression deregulation (EXP), and selected gene mutations in 188 primary BC samples. These analyses identified luminal B-specific candidate genes.

## Materials and Methods

### Ethics statement

The study was approved by our institutional review board: the “Comité d'Orientation Stratégique” of the Institut Paoli Calmettes (IPC) (Marseille, France). Each patient gave a written informed consent for research use.

### Breast cancer samples

Pre-treatment tumor tissues were collected from 188 patients with invasive adenocarcinomas. Patients underwent surgical biopsies or initial surgery at the Institut Paoli-Calmettes between 1987 and 2007. The main histoclinical, biological and subtype characteristics were established for the 188 BCs as described [12], [17]–[19]. They are listed in **Table S1A** and illustrated in **Figure S1**.

### Gene profiling and data analysis

DNA and RNA were extracted as previously described [12], [17]–[19] and controled on Agilent Bioanalyzer (Agilent Technologies, Massy, France). Genomic profiles of the 188 BCs were established by using array-comparative genomic hybridization (aCGH) onto high-resolution 244K CGH microarrays (Hu-244A, Agilent Technologies, Massy, France). A pool of 13 normal male DNA was used as reference. Gene expression data from the same 188 BCs and 4 normal breast (NB) samples, which represented 1 pool of 4 samples from 4 women, and 3 commercial pools of respectively 1, 2 and 4 normal breast RNA (Clontech, Palo Alto, CA), were obtained using whole-genome DNA microarrays (HG-U133 Plus 2.0, Affymetrix). Both approaches and analysis methods have been used in our previous studies [12], [17], [18]. All probes for aCGH, gene expression and DNA promoters methylation analyses were mapped according to the hg18/NCBI human genome mapping database (build 36) to homogeneously integrate the data. The aCGH, gene expression, methylation data, as well as the integrated CNA/gene expression and DPMA/gene expression analyses are illustrated by pipelines (**Figures S2 and S3**, respectively).

Validation and prognostic impact of candidate genes were evaluated in a large public series of BC samples. Thirty-six data sets, including a total of 5,765 non-redundant samples, were collected from public database i.e. Gene Expression Omnibus (GEO/NCBI), Array Express (EBI) and authors' websites (**Table S1B**). Raw data from each study were normalized using quantile normalization, and log2-transformed. The intrinsic molecular subtypes of each tumor sets were defined using Hu single sample predictor (SSP) [4]. To be comparable across data sets, each gene expression levels were standardized within each data set using luminal A population as reference.

The data (experiment called “Candidate luminal B breast cancer genes identified by genome, gene expression and DNA methylation profiling”) are publicly available (ArrayExpress repository ref ID: E-MTAB-1861).

### DNA promoter methylation profiling and data analysis

We captured the methylated DNA of 117 (109 tumors+8 NB) samples by using the MethylMiner Methylated DNA Enrichment Kit (Invitrogen). Genome-wide DNA-methylation analysis was done on custom [A-MEXP-2178 (arrayexpress)] human promoter arrays 2×400K (Agilent Technologies, Massy, France) using the MethylMiner Methylated DNA Enrichment Kit (Invitrogen) [36]. Over 414,000 probes cover promoter regions approximately −3 kb to +3 kb relative to transcription start sites (TSSs) with a resolution of 280 bp in average. Scanning was done with Agilent Autofocus Dynamic Scanner (G2565C, Agilent Technologies). Raw data were obtained from Feature extraction 10.7.3 software (Agilent Technologies). Probes not mapped to exact positions in the genome as well as those under the background signal were removed with the control probes. The final dataset contained 326,350 unique probes covering 18,297 promoter regions according to the hg18/NCBI human genome mapping database (build 36). The M (Log_2_Red-Log_2_Green) values of each probe on the array were then obtained and normalized according to their GC content. Then, inter-array quantile normalization was done for the correction of distribution differences among experiments.

To estimate the global methylation level for a given gene in one sample, we computed a methylation score based on the sum of frequency of probes with a M value greater than zero combined with its amplitude and frequency of probes with a M value less than zero combined with its amplitude. Clustering was done with the Cluster program using Pearson correlation as similarity metrics and average linkage clustering. Results were displayed using TreeView program.

### Gene mutations

Polymerase chain reaction (PCR) and direct sequencing were done using standard conditions with gene-specific primers designed to amplify coding sequence of *ARID1A, ASXL1, FOXO3, L3MBTL4, MAP2K4, PIK3CA, RUNX1, RUNX3* (**Table S1C**). Most PCR amplifications were done in a total volume of 25 µl PCR mix containing at least 10 ng template DNA, Taq buffer, 200 µmol of each deoxynucleotide triphosphate, 20 pmol of each primer and 1 unit of appropriated Taq polymerase (**Table S1C**). PCR products were purified using Millipore plate MSNU030. The purified PCR products (2 µl) were used for sequencing using the Big Dye terminator v1.1 kit (Applied Biosystems, Courtaboeuf, France). After G50 purification, sequences were loaded on an ABI 3130XL automat (Applied Biosystems). The sequence data files were analyzed using the Seqscape software and all mutations were confirmed on an independent PCR product.

*TP53* mutation status was determined by a yeast functional assay [37], [38].

### Statistical analyses

Correlations between sample groups and histoclinical factors were calculated with the Fisher's exact test for qualitative variables with discrete categories, and the Mann-Whitney test for continuous variables. Metastasis-free survival (MFS) was calculated from the date of diagnosis until the date of first metastatic relapse. Survivals were calculated using the Kaplan-Meier method and curves were compared with the log-rank test. Stratification into high-risk and low-risk groups was based on relative risk defined by Cox model using the natural threshold of 1. Univariate and multivariate survival analyses were done using the Cox regression model (Wald test). Variables tested in univariate analyses included patients' age at time of diagnosis (≤50 years *vs* >50), pathological tumor size (pT: pT1 *vs* pT2-3), pathological axillary lymph node status (pN: negative *vs* positive), pathological grade (I *vs* 2–3), histological type, delivery of hormone therapy and chemotherapy, immunohistochemical (IHC) ERBB2 status (negative *vs* positive), molecular subtypes and *RECQL4* expression (continuous value). Variables with a p-value<0.05 in univariate analysis were tested in multivariate analysis. All statistical tests were two-sided at the 5% level of significance. Analyses were done using R software (2.14.2) and associated packages. We followed the reporting REcommendations for tumor MARKer prognostic studies (REMARK criteria) [39].

Luminal B CNAs landscape was drawn with the Circos software [40] and significant mutual exclusive and co-occurring CNAs (FDR<0.05) were identified by DRP analysis [41].

In the meta-analysis of six recent NGS studies including 875 breast tumors, co-occurring and mutually exclusive gene mutations were identified by using a method previously reported [42].

## Results

### Genomic characterization of 188 BC samples

We first describe the results on the whole set of 188 tumors before addressing the specific question of the luminal B cases. The 188 samples were first profiled using whole-genome gene expression microarrays. **Figure S1** shows the hierarchical clustering of samples based on the expression of 13,031 probe sets. Samples were sorted into four major clusters, which strongly correlated with histoclinical features (grade, IHC data) and molecular subtypes. The 188 cases (**Table S1A**) included 54 basal, 64 luminal A, 32 luminal B, 16 ERBB2 and 22 normal-like cases [4] (**Table S1D**).

High-resolution aCGH profiles were established for the 188 samples. High and low level CNAs (amplifications or homozygous losses and gains or hemizygous losses, respectively) were identified. As previously reported [8], the three most frequently gained regions were on the 1q, 8q and 17q chromosomal arms whereas the most frequently lost regions were on 8p, 11q and 16q. The median percentage of probe sets displaying a CNA in a sample was 41%, with a great variability between samples (range 0.05–80%). As expected, this percentage was higher in grade 3 tumors (52%) than in grade 1 tumors (17%; p = 1.92×10^−7^; Mann-Whitney test). A total of 52 cases (28%) were “simplex”, 81 (43%) “complex sawtooth”, and 55 (29%) “complex firestorm” (**Table S1A**). As expected, only 11% of grade 3 tumors were “simplex”, whereas 89% were “complex” and conversely, 53% of grade 1 tumors were “simplex” and 47% “complex”. The highest proportion of simplex patterns was observed in luminal A tumors (58%) whereas the highest proportion of complex patterns was found in ERBB2 (100%), luminal B and basal (91% for both) cases (**Fig. S4**).

### Copy number aberrations in luminal B tumors

We next focused on the CNAs found in luminal B tumors. The luminal B subtype shows both common and specific alterations [7], [9], [16], [19]. To identify the latter we did a supervised analysis comparing CNAs observed in luminal B to those found in each of the other subtypes (**Fig. S5** and **Tables S2A–F**).

The luminal B/luminal A comparison showed that 8p11-12 amplification and gains of distinct regions (i.e. 7p11.2-22.1, 7q11.21-36.3, 8p11-12, 8q, 11q13-14, 12, 14q12-23, 17q11-25.3, 18q12.1, 20p11.21-12.3 and 20q with a frequency ≥30%) are more frequent in luminal B samples (Fisher's exact test; FDR<0.05) (**Fig. S5**, **Tables S2A, B**). CNAs associated with gains or amplification targeted a total of 3,364 genes (**Table S2B**). Genes amplified in luminal B included the 8p12 genes *ZNF703*, *SPFH2*, *PROSC*, *GPR124*, *BRF2*, *RAB11FIP1*, *GOT1L1*, *ADRB3*, *EIF4EBP1*, *ASH2L*, *STAR* and *LSM1*.

The luminal B/basal comparison showed that gains and losses of distinct regions (including 5q11.1, 8p11-12, 8q13.2-21.3, 11q13.3, 12q12-24.23, 14q21.3-24.23, 16p11.2-13.3, 17q, 20q11.23-q13.33 and 6q, 9p22-p24, 13q34, and 18p11.31-11.32 regional gains and losses with a frequency ≥30%, respectively) are more frequent in luminal B samples (Fisher's exact test; FDR<0.05) (**Fig. S5**, **Tables S2C,D**). Gains and losses targeted 1,091 and 3,339 genes, respectively (**Table S2D**). The comparison with the ERBB2 subtype did not distinguish regions with a different CNA frequency, perhaps because of the low number of samples.

**Figure 1:**
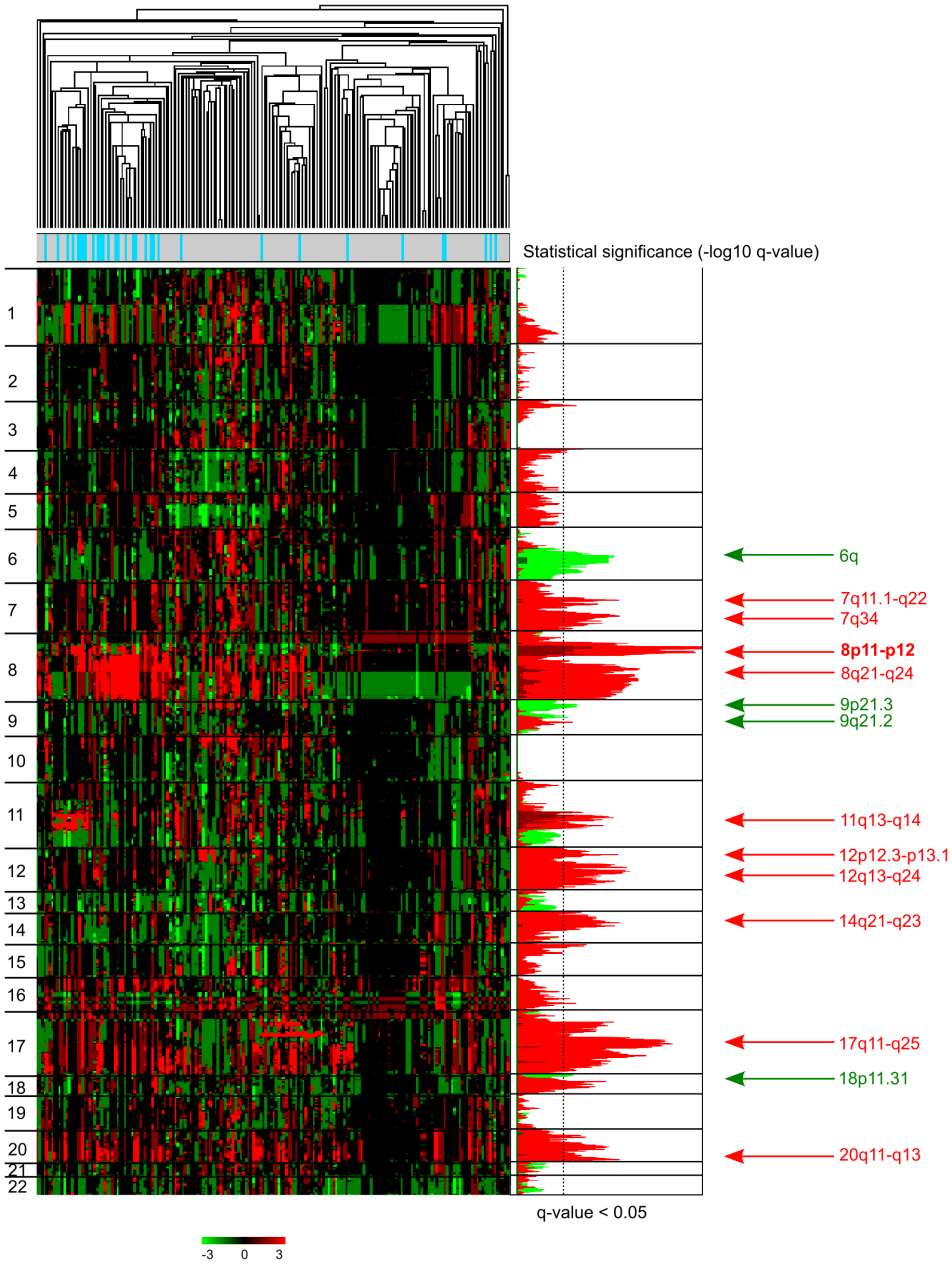
Specific regions targeted by CNAs in luminal B BCs. Genomic profiles were established for 188 breast tumors (32 luminal B and 156 non luminal B). On the left is shown a hierarchical clustering of genome copy number profiles measured by aCGH on 24,907 probes or groups of probes (without X and Y). Red indicates increased copy number and green indicates decreased copy number. To the left are indicated chromosome locations with chromosome 1pter to the top and 22qter to the bottom. Next on the right, significant copy number amplifications (dark red), gains (red) and losses (green) observed in luminal B compared to non-luminal B tumors (Fisher's exact test), are plotted as a function of chromosome location. Only amplification, gains, and losses associated with luminal B tumors are shown (Fisher's exact test; FDR<0.05) for each chromosome. In addition to the previously reported 8p11-12, 11q13 amplifications, 17q, 20q gains and 18p losses we previously reported (16,20), other luminal B CNAs include 7q11.22, 7q34, 8p11.21-p12, 8q21.12-q24.23, 11q13.3-q14.1, 12p12.3-p13.1, 12q13.11-q24.11, 14q21.1-q23.1, 17q11.1-q25.1, 20q11.23-q13.33 gains and 6q14.1-q24.2, 9p21.3-p24.3, 9q21.2, 18p11.31-p11.32 losses.

The luminal B/non-luminal B comparison (non-luminal B includes luminal A, ERBB2, basal, normal-like) (**Fig. 1**) showed that 8p11-p12 and 11q13.1-q13.4 amplifications, gains of distinct regions (including 7q11.22-q34, 8p11.21-p12, 8q21.12-q24.23, 11q13.3-q14.1, 12p12.3-p13.1, 12q13.11-q24.11, 14q21.1-q23.1, 17q11.1-q25.1, 20q11.23-q13.33 gains and 6q14.1-q24.2, 9p21.3-p24.3, 9q21.2, 18p11.31-p11.32 losses with a frequency ≥30%, respectively) are more frequent in luminal B samples (Fisher's exact test; FDR<0.05) (**Fig. 1**, **Tables S2E,F**). Amplification, gains and losses targeted 122, 2,541 and 277 genes, respectively (**Table S2F**). The most significant amplified genes associated with luminal B included again the 8p12 genes *ZNF703*, *SPFH2*, *PROSC*, *GPR124*, *BRF2*, *RAB11FIP1*, *GOT1L1*, *ADRB3*, *EIF4EBP1*, *ASH2L*, *STAR* and *LSM1* (34%; Fisher test, p<9.5×10^−6^, FDR<8.1×10^−3^) and the 11q13 genes *CCND1*, *ORAOV1, FGF19, FGF4, FGF3* (38%; Fisher test, p<1×10^−4^, FDR<0.05) (**Table S2E**). Many of the 277 lost genes were located in chromosome arm 6q with a frequency comprised between 50% and 66% (**Table S2E**). The most significant losses were associated with *C60RF167*/*MMS22L* (6q16.1) and *MCHR2*, *SIM1*, *ASCC3* (6q16.3) (66%; Fisher test, p = 7.75×10^−7^, FDR = 1.93×10^−3^). They were 3.3 times more frequent in luminal B than in non-luminal B tumors. Genomic profiles showed various 6q regional losses as well as rare homozygous deletions and small deleted regions targeting *MLLT4*, *ARID1B*, *PARK2*, *FOXO3, UFL1/NLBP, ASCC3* genes (**Fig. S6**), suggesting the existence of several potential TSGs within 6q. Chromosomal regions 9p21.3-24.3 and 9q21.2 were targeted by losses with a frequency comprised between 50% and 56%, respectively (**Fig. S7** and **Table S2E**). These events were twice more frequent in luminal B than in non-luminal B tumors (56%, Fisher test, p<1 10^−3^, FDR<0.05). *MTAP*, *CDKN2A* and *CDKN2B* were the most frequently 9p deleted genes in luminal B samples. Chromosomal region 18p11.31-11.32 was targeted by copy number losses with a frequency comprised between 53% and 56% (**Table S2E**). These losses were almost twice more frequent in luminal B than in non-luminal B tumors (56%, Fisher test, p<2×10^−3^, FDR<0.05). Noticeably, genes coding for TP53 repressor *PRDM1* (6q21) and TP53 effector *PERP* (6q23.3) were among the genes with loss frequencies associated with the luminal B subtype (**Table S2E**). Conversely, gains of *TP53INP1* (8q22.1) (81%, Fisher test, p<9×10^−4^, FDR<0.05), *TP53I13* (17q11.2) (50%, Fisher test, p<5×10^−4^, FDR<0.05), *TP53INP2* (20q11.22) (56%, Fisher test, p<5×10^−3^, FDR<0.05) and *TP53RK* (20q13.12) (56%, Fisher test, p<1×10^−2^, FDR<0.05) were associated with the luminal B subtype (**Table S2E**). This suggests that a TP53-associated pathway may play a role in luminal B tumors.

### Integrated comparative analysis and luminal B candidate genes

We compared the degree of CNA-driven RNA up or downregulation in 32 luminal B (i) vs 64 luminal A, (ii) vs 54 basal, and (iii) vs 156 pooled non-luminal B tumors, by analyzing the 13,127 genes common to the two platforms (aCGH Agilent and Hu233 2.0 plus Affymetrix) and retained after filtering based on the expression level. From these supervised analyses (**Tables S2A–F**), 160, 148 and 307 genes had an expression level that varied according to CNA (Mann-Whitney; FDR<0,05) after luminal B/luminal A (**Table S3A**), luminal B/basal (**Table S3C**), and luminal B/non-luminal B (**Table S3E**) comparison, respectively. Of these, 138, 132, and 251 genes had a deregulated gene expression in relation with CNA specifically associated with the luminal B subtype (t test, FDR<0.05) after luminal B/luminal A (**Table S3B**), luminal B/basal (**Table S3D**), and luminal B/non-luminal B (**Table S3F**) comparison, respectively. These specific luminal B candidate genes were qualified as potential oncogenes or TSGs if they presented up or downregulated expression in relation with amplification/gain or losses, respectively, as previously described [12], [17], [18]. Overall, out of the 337 candidates identified, 66% (221) were found in the TCGA data [28], deregulated in relation with their copy number alterations. (**Tables S3B**, **3D** and **S3F**, respectively).

**Figure 2:**
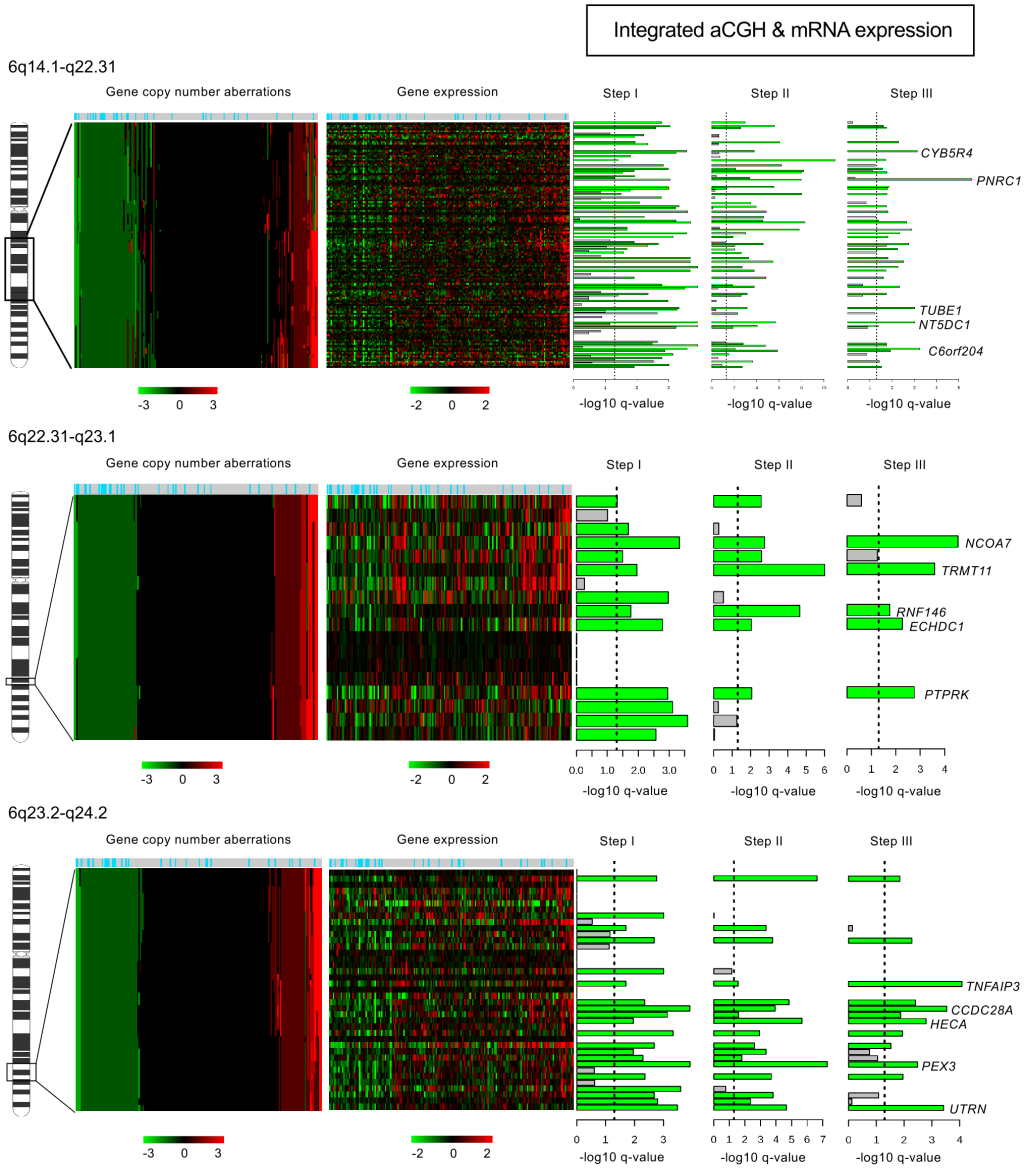
Correlation between gene expression and genome alterations on the 6q regions. Genomic and gene expression profiles were established for 188 breast tumors (32 luminal B and 156 non luminal B identified at the top by blue and grey boxes, respectively) in three 6q regions: 6q14.1-q22.31 (top), 6q22.31-q23.1 (middle) and 6q23.2-q24.2 (bottom). For each region, heatmaps for genome copy number and gene expression profiles are consecutively drawn. Genome copy number was measured by aCGH on probes or groups of probes spanning each of these regions. Red indicates increased copy number and green indicates decreased copy number. In the heatmap tumors are organized from the tumor that presented the most copy number losses to the tumor that exhibited the most copy number gains. The next heatmap was established with the expression of the independent genes located on the corresponding 6q region and profiled in the same 188 tumors similarly organized. For gene copy number and gene expression heatmaps, we used color scale limits from −3 to +3 and −2 to +2, respectively. Next to the right, are plotted genes successively selected by steps I, II and III of the integrated analysis “aCGH & mRNA expression” as defined by the work pipeline (Figure S2). Grey and green lines correspond to rejected and selected genes, respectively. Among genes with an expression level that varied according to CNAs, we retained genes showed significant differences (vertical line) in copy number loss correlated with downregulated expression in luminal B compared to non-luminal B tumors. They were qualified as potential TSGs. For each region, only the first five most significant are listed. *PNRC1*, *NCOA7* and *TNFAIP3* genes were the most significant candidate TSGs for the 6q14.1-q22.31 (top), 6q22.31-q23.1 (middle) and 6q23.2-q24.2 (bottom) regions, respectively.

Among the 251 luminal B candidate genes of the luminal B/non-luminal B comparison (**Table S3F**), 189 and 62 were potential oncogenes and TSGs, respectively. By order of significance, *ZNF703, CCND1, ORAOV1, BRF2, RAB11FIP1, LSM1, PPAPDC1B, ASH2L, DDHD2, AP3M2, VDAC3, FGFR1, EIF4EBP1, MYST3*, and *TM2D2* were overexpressed in relation with their amplification (**Fig. S8**). Among the 62 TSG candidates, 53, 6, 2 and 1 were located on 6q (**Fig. 2**), 9p21.3-p24.3, 18p11.31 and 9q21.2 regions, respectively.

A total of 39 luminal B candidate oncogenes were identified from luminal B/normal-like comparisons (**Table S3G**). No luminal B candidate was identified from the luminal B/ERBB2 comparison.

Among luminal B candidates defined from the different comparisons, 7 (BRCA1, CCND1, COX6C, EZH2, FGFR1, MSI2, RECQL4) code for potentially druggable proteins [43], and 11 (*BIRC5, CITED2, FAM82B, FOXM1*, GPR172A, NHERF1, RECQL4, SLC39A4, SQLE, UBE2C, YWHAZ) are associated with endocrine resistance (**Table S3G**) [33], [34], [44].

A total of 34, 47 and 2 candidate genes were identified as specific of luminal B tumors when compared to luminal A, basal, and normal-like tumors (**Tables S3H–J**), respectively. These different repertoires of candidate genes could help identify pathways and mechanisms either specifically affected in luminal B tumors or shared by the major subtypes.

Interestingly, 101 luminal B candidate oncogenes were common to both luminal B *vs* luminal A and luminal B *vs* non-luminal B comparisons (**Table S3K**). This “core” of luminal B oncogenes was mainly associated with the cell cycle and proliferation (p<0.05; FDR<0.25) (**Table S3L**). They included 14, 4, 37, 8, 23 and 10 genes from amplified 8p11-p12, 11q13, and gained 8q13-q24.3, 12q, 17q11.2-q25.1 and 20q11-q13 regions. Among them, *AP3M2, ASH2L, BRF2, DDHD2, FGFR1, LETM2, LSM1, PPAPDC1B, RAB11FIP1, ZNF703* (8p12), *TPD52* (8q21.13), and *DCAF7/WDR68* (17q23.3) have been described as potential BC genes [6], [12], [19], [45]–[47]. In a large public series of 5,765 BCs, 100 (99%) of the candidate oncogenes included in this “luminal B core” were validated at the gene expression level in both comparisons (**Table S3M**). The overexpression of 67 of them was associated with poor metastatic-free survival (MFS) in luminal tumors (p<0.05; FDR<0.25). While several data show that loss of PR expression is an important predictor of poor patient outcome in ER+ BCs [48], [49], 94% (61/67) of our luminal B candidates were pertinent predictor independently of the PR status (**Table S3M**).

**Figure 3:**
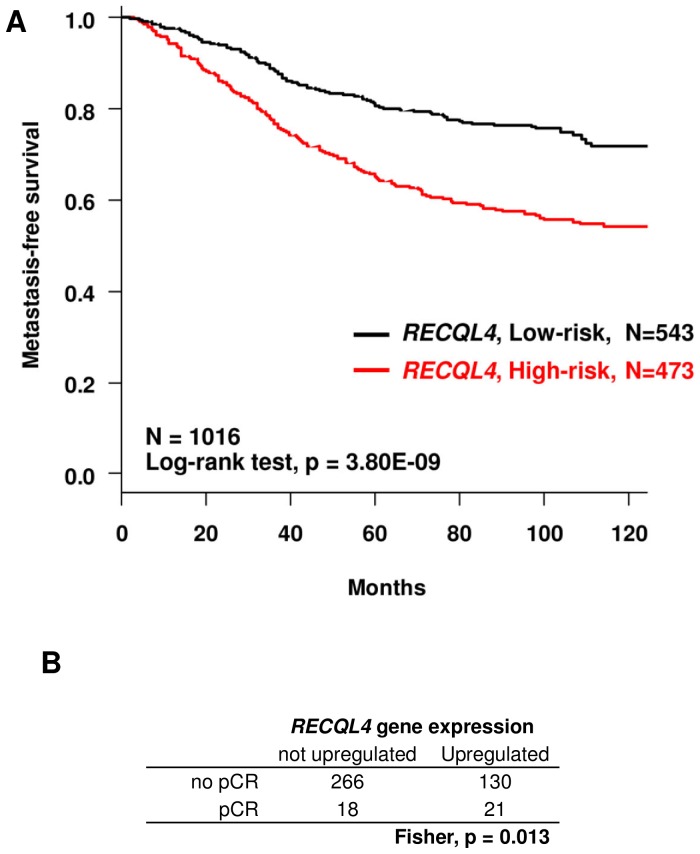
Kaplan-Meier MFS curves and pathological clinical response in luminal BCs according to *RECQL4* mRNA expression. **A**-Kaplan–Meier survival curves were drawn according to *RECQL4* gene expression status of 1,016 luminal BCs established from a large public series of 5,765 BCs (**Table S1B**). Stratification into high-risk (red curve) and low-risk (black curve) groups were based on relative risk defined by the Cox model using the natural threshold of 1. *RECQL4* gene expression is associated with MFS (p = 3.8010^−9^). **B** - From the same large public series, 924 BCs were informed for the pathological response (**Table S1B**). The *RECQL4* overexpression was associated with a better pathological response within the group including both luminal A and B tumors (N = 435) (Fisher, p = 0.013).

*RECQL4* was the most significant. Corresponding Kaplan-Meier MFS curves are shown in **Figure 3A** (p = 3.80×10^−9^). Interestingly, concordant results were obtained with another referent molecular classifier i.e. PAM50 SSP [50] (**Table S3M**). Indeed, the definition of luminal B tumors is highly variable due to their molecular heterogeneity, making their assignment in the luminal B subtype non-reproducible by different gene expression signatures [51].This suggests that the robustness of our candidates is not impacted by the choice of the classifier [4], [50].

A high *RECQL4* gene expression level has recently been reported to confer proliferation advantage and survival to breast cancer cells [50]. We observed that *RECQL4* overexpression was associated with *MDM2* overexpression (t-test p = 2.75×10^−2^) as well as with mutated *TP53* or/and overexpressed *MDM2* gene status (t-test p = 1,73×10^−20^) in the independent public TCGA data set [28] (data not shown). This might highlight high genomic instability and DNA repair perturbations in such tumors.

In multivariate analysis including the other histoclinical prognostic features, *RECQL4* expression remained significant for MFS (p = 3.4×10^−2^), with pT and molecular subtypes (**Table 1**). The clinical response study in regard to *RECQL4* gene expression in 924 BCs (included in the large public series, see **Table S1B**) showed as expected that luminal B tumors have a better pathological response to chemotherapy (Fisher, p = 3.04×10^−4^) than luminal A tumors. Moreover, *RECQL4* overexpression was associated with a better pathological response in the group including both luminal A and B tumors (Fisher, p = 0.013) (**Figure 3B**) but not within separated luminal subgroups (data not shown).

**Table 1:** Uni- and multivariate analysis of MFS in the luminal BC public data.

		Univariate			Multivariate		
		N	HR [95CI]	p	N	HR [95CI]	p
Age	>50 *vs* < = 50 y	512	1.03 [0.73–1.46]	0,87			
**pT**	pT2-3 *vs* pT1	556	1.76 [1.25–2.48]	**1,30E-03**	509	1.51 [1.05–2.18]	**2,65E-02**
**pN**	pos *vs* neg	829	1.21 [0.9–1.63]	0,22			
**SBR grade**	2–3 *vs* 1	650	2.72 [1.69–4.4]	**4,22E-05**	509	1.39 [0.82–2.34]	0,22
**Histology** **	ILC *vs* IDC	128	1.45 [0.59–3.56]	0,41			
	other *vs* IDC	128	0.78 [0.18–3.29]	0,73			
**Hormone therapy**	Yes *vs* No	746	0.64 [0.45–0.93]	**2,01E-02**	509	0.68 [0.41–1.12]	0,13
**Chemotherapy**	Yes *vs* No	749	1.08 [0.71–1.66]	0,71			
**ERBB2 IHC status**	pos *vs* neg	148	1.19 [0.36–3.91]	0,77			
**Molecular subtype** *	LumB *vs* lumA	1016	2.07 [1.67–2.57]	**3,99E-11**	509	2.53 [1.66–3.87]	**1,66E-05**
***RECQL4***		1016	1.55 [1.37–1.75]	**1,44E-12**	509	1.33 [1.02–1.73]	**3,40E-02**

as previously defined [4].

IDC: infiltrated ductal carcinomas; ILC: infiltrated lobular carcinomas.

We further identified 4 mutually exclusive CNA pairs (*LYZ* gains/6q25-qter losses, *RUNX1* loss/6q22.1-q24.3 losses, *RUNX1* loss/*RUNX3* loss and *RUNX1* loss/6q14.1-q21 losses) and 215 co-occurring CNAs in luminal B (FDR<0.05) (**Fig. S9, Table S3N**). The mutually exclusive CNA pairs suggest that these altered genes and/or regions could participate in the same pathways.

### DNA methylation profiles of 109 BCs

**Figure 4:**
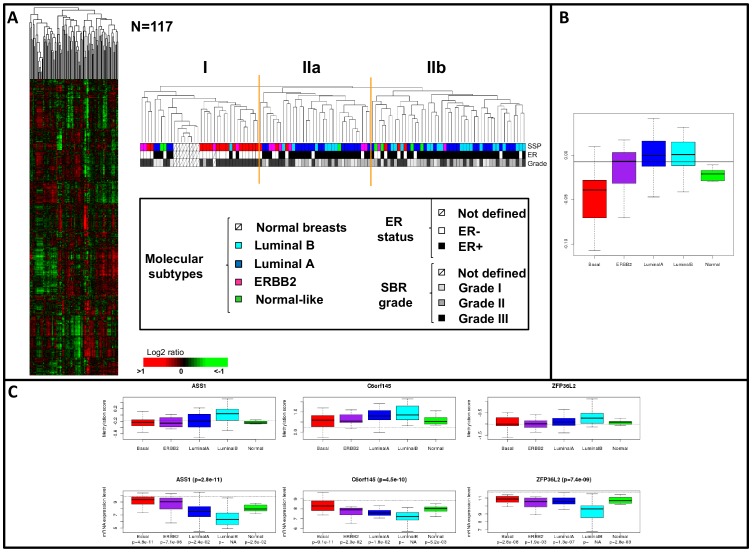
DNA Methylation promoter profiles in breast cancers. **A**- Hierarchical clustering established with the most variant methylation scores observed in 5,492 gene promoters (SD>0.3) in 109 tumors samples and 8 normal breast tissues samples. Each row of the data matrix represents a gene promoter and each column represents a sample. DNA methylation variations are depicted according to the color scale shown at the bottom. Red indicates increased DNA methylation score and green indicates decreased DNA methylation score. The dendrogram of samples (above matrixes) represents overall similarities in DNA methylation profiles and is zoomed to the right. Three groups of tumor samples (I, IIa and IIb) are associated with various DNA methylation patterns and delimited by orange vertical lines. Below the dendrogram are some histoclinical and molecular features of the samples: from top to bottom, intrinsic molecular subtypes^4^, IHC ER status and SBR grade. Color legends for the various features are illustrated below. **B** - The median methylation levels of the 4,545 subtype-associated genes were highest in the luminal and ERRB2 subtypes and lowest in the basal subtype (p = 6.24 10^−12^). **C** - Compared to the other molecular subtypes, the DNA methylation levels (three top panels) of *ASS1*, *C6ORF145* and *ZFP36L2* gene promoters and their mRNA expression (three bottom panels) were higher (**Table S4E**) and lower in the luminal B BCs, respectively.

The hierarchical clustering established with the most variant methylation scores observed in 5,492 promoters (SD>0.3) classified the 109 BC and 8 NB samples into clusters associated with different DNA methylation patterns (**Fig. 4A**) variably associated with ER and PR expression (p = 5.6×10^−7^ and p = 2.26×10^−7^), SBR grade, molecular subtype (p = 5×10^−4^), and *TP53* status (p = 1.2×10^−3^) (**Table S4A**). The 8 NB samples and most of the ER- tumors were included in cluster I while ER+ tumors were mainly distributed in cluster II. Most of the basal tumors were included in cluster I whereas cluster II contained most of the luminal A and luminal B tumors. ERBB2 tumors were distributed in the two clusters probably because ERBB2 tumors are heterogeneous for ER status [17]. These different profiles were consistent with recently reported BC methylation patterns [21]–[23], which pointed to a possible relationship between DNA methylation and ER status.

### Validation of methylation results

The supervised analysis comparing methylation score data of ER+ and ER- tumors identified 3,484 gene promoters with methylation differences between the two groups (**Table S4B**) (t test, FDR<0.05). Among them, 1,753 gene promoters (including those of *APC*, *CAV1*, *CCND2*, *CDCA7*, *CDH3*, *CDKN2A*, *CDKN2B*, *HEY2*, *RASSF1, RECK*) had a DNA methylation level higher in the ER+ group (t-test, FDR<0.05). Conversely, 1,731 gene promoters (including those of *BCL2*, *ESR1*, *HSD17B4*, *PISD*, *WNK4*) had a higher level of methylation in the ER- group (FDR<0.05). These data are in agreement with those reported on Illumina® platform [20], [21], [23], [24]. The strong correlation between DNA methylation data of *RASSF1* gene promoter established by EpiTyper and promoter array approaches further validated our method (**Results and References S1, Fig. S10, S11**).

### DNA methylation associated with breast cancer molecular subtypes

Using the ANOVA method, we identified 4,545 gene promoters with a DNA methylation level different in at least one subtype among the five major ones (**Table S4C**). They included several genes previously reported differentially methylated in BC subtypes and particularly in luminal and basal (or ER+ and ER−) tumors (**Table S4C**). The median methylation levels of these 4,545 subtype-associated genes were highest in the luminal and ERBB2 subtypes and lowest in the basal subtype (p = 6.24×10^−12^) (**Fig. 4B**).

Tukey's range test integrating ten supervised analyses was applied to distinguish gene promoters exhibiting a DNA methylation level different in only one subtype as compared to the others (FDR≤0.05) (**Table S4C**). A total of 375 and 431 promoters had a methylation level higher and lower in the luminal B tumors than in the other subtypes, respectively. Among them, 265 and 295 had a methylation level higher and lower than in NB tissues (t test, FDR<0.05). This analysis was extended to the other subtypes (**Results and References S1**).

### Molecular subtypes and specific deregulated gene expression in relation with the DNA methylation level variation

Among the 4,545 promoters with a DNA methylation level different in at least one subtype (**Table S4C**), only 459 genes presented an associated mRNA deregulation (correlation<−0.40) (**Table S4D**). These genes are listed with their CNA status in **Table S4E**. The presence of CpG islands was observed in 62.5% of them. In the luminal B cases, among the 560 promoters associated with a significant DNA methylation variation, 46 corresponding genes presented a deregulated expression (24 and 22 were down and upregulated, respectively) (correlation<−0.40) (**Table S4E**). 52% (24 genes) were found significantly deregulated in relation with their level of DNA methylation in the TCGA data [28] (**Table S4E**).

Among them, high DNA methylation level targeted promoters of *ASS1*, *CITED4*, *DCR1*, *FAM78A*, *FBXO32*, *SAMD9L*, *SP100*, *STAT5A* and *ZFP36L2* genes, previously reported as TSGs or associated with tumor progression (**Table S4E**). Only lower *ASS1*, *C6ORF145/PXDC1*, *ZFP36L2* and higher *C3ORF67, C12ORF60, H2AFJ, RAB11FIP1* gene expression were observed in luminal B compared to the other subtypes (t test, p<0.05) (**Fig. 4C** **and S12A**). The other subtypes were also analyzed (**Results and References S1, Table S4E, Fig. S12B–S12D1–D4**). Overall, out of the 168 candidates identified in our analysis, 54% (90 candidate genes) were found in the TCGA data [28] significantly deregulated in relation with their level of DNA methylation (**Table S4E**).

### Subtype-specific candidates presenting gene expression deregulation in relation with CNA and with DNA methylation aberrations

We integrated genomic, gene expression and DNA methylation profiles to identify subtype-specific candidate genes presenting expression deregulation in relation with CNA and with DNA promoter methylation aberrations (**Table S4F**).

In the luminal B cases, no gene was downregulated in relation with both copy number loss and with high methylation (**Table S4F**). Conversely, 8 genes were upregulated in relation with copy number gain or amplification and with low DNA methylation (**Table S4F**). *C12ORF60* and two previously reported oncogenes, *H2AFJ* and *RAB11FIP1/RCP* were the most significantly upregulated (t-test, p<0.05) (**Fig. S12A**). Except for *TPD52* and *C12orf60*, 75% were upregulated in relation with copy number gain or amplification and with low DNA methylation in the TCGA data [28]. The other subtypes were also analyzed (**Results and References S1, Table S4F, Fig. S12B–S12D1–D4**).

### Mutations in candidate genes

Based on both previous studies and our observations of potential driver CNAs and mutations in luminal B BCs [16], [27]–[31], [53] (**Table S5A**), we searched for mutations in *ARID1A*, *ASXL1*, *FOXO3*, *L3MBTL4*, *MAP2K4*, *PIK3CA*, *RUNX1*, *RUNX3* and *TP53* genes in a large set of 390 BCs, including a part of our panel (181/188 BCs) (**Tables S5B–C**). Mutations (including frameshift, nonsense, missense mutations) targeted in decreasing frequency order *TP53* (46.3%), *PIK3CA* (23.3%), *RUNX3* (9.1%), *FOXO3* (8.6%), *RUNX1* (4.7%), *ARID1A* (3.7%), *MAP2K4* (3.3%), *L3MBTL4* (2.5%), and *ASXL1* (2.2%) genes (**Table S5C**). *ARID1A* and *L3MBTL4* mutations were previously reported [16], [53]. Mutations in *TP53, PIK3CA, MAP2K4* and *RUNX1* genes were previously reported as driver mutations [27], [29]–[31]. We verified that the p.A147Gfs*13 and p.S295Cfs*304 *RUNX1* and p.S588X *FOXO3* mutations were acquired (**Fig. S13, S14**, respectively). We could not confirm that the other missense mutations were similarly somatic. However, they were not included as SNP or missenses in NCBI db SNP build 131.

**Figure 5:**
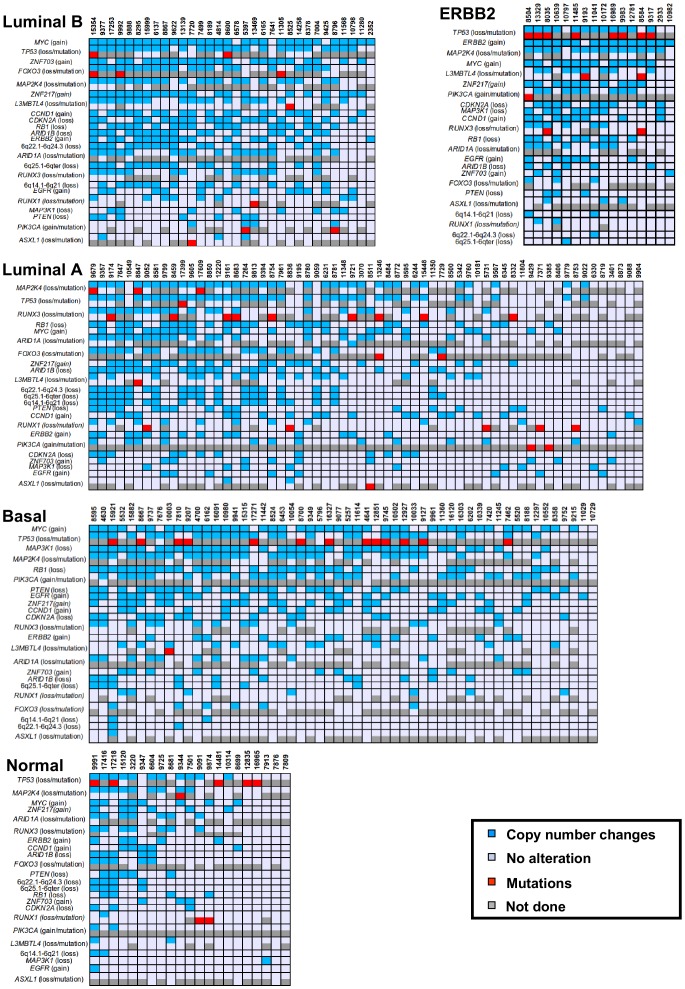
Landscape of specific regions/genes in the 188 breast tumors. The most significant amplifications/gains, losses and mutations in four major subtypes are shown.

As illustrated for *RUNX1* (**Fig. S15**), some genes can be targeted by different alterations (CNAs, mutations) with frequencies that vary in the major subtypes (**Table S5C**). In the luminal B cases, we identified mutations of *FOXO3* (23.8%), *TP53* (23.1%), *PIK3CA* (16.7%), *RUNX3* (8.5%), *ASXL1* (7.1%), *MAP2K4* (6.1%), *RUNX1* (4.9%) and *L3MBTL4* (2.1%) genes We found that only *FOXO3* mutations were specifically associated with this subtype (Fisher test, FDR<0.05 and odd ratio>1) (**Table S5C**). No *ARID1A* mutation was observed. The mutations existed in the luminal B genomic context including *ZNF703*, *MYC*, *CCND1* and *ZNF217* gains/amplifications and *FOXO3*, *CDKN2A*, *L3MBTL4* and *ARID1B* losses (Fisher test, FDR<0.05 and odd ratio>1) (**Table S5C**). Only *RUNX3* and *TP53* mutations were significantly found in luminal A and basal tumors, respectively (t-test, FDR<0.05 and odd ratio>1) (**Table S5C**). *PIK3CA*, *EGFR*, *MYC* gains/amplifications, *MAP3K1* losses and *ERBB2* amplification reflected genomic contexts associated with basal and ERBB2 molecular subtypes, respectively. **Figure 5** illustrates the presence of CNAs and/or mutations targeting some of these genes in each of the breast tumor samples constituting our panel and classified by molecular subtype.

### Meta-analysis of six recent NGS studies in 875 breast tumors

To include our results in a more global context we did a meta-analysis of whole genome/exome sequence data generated from a total of 875 BC samples (“NGS samples”) in six 2012 studies (**Table S6A**). A total of 35,603 somatic mutations (including in/del, nonsense, missense mutations) were identified in 13,793 genes (**Table S6B**). In a decreasing frequency order, *TP53*, *PIK3CA*, *TTN*, *GATA3*, *MLL3*, *MAP3K1*, *MUC16*, *CDH1*, *SYNE1* and *USH2A* were the top ten mutated genes in the NGS samples; the frequencies of somatic mutations in *TP53, PIK3CA*, *RUNX1, ARID1A*, *MAP2K4*, *L3MBTL4* and *ASXL1* were 34%, 31%, 2.4%, 2.1%, 3.4%, 0.3% and 0.6%, respectively.

To identify co-occurring and mutually exclusive gene mutations, we retained genes targeted by more than 15 mutations in the 875 NGS samples. From 70 such genes, we identified 315 co-occurring and 8 mutually exclusive gene mutations (p<0.05) (**Tables S6C**). Concomitant mutations of *MAP3K1/PIK3CA*, *CDH1/PIK3CA*, *MAP2K4/PIK3CA* were among the most frequently observed (>15 mutations). *MAP3K1/TP53, GATA3/TP53, CDH1/TP53, AKT1/PIK3CA, MAP2K4/TP53* and *CDH1/GATA3* mutations were mutually exclusive.

In these NGS studies, only 602 samples were subtyped (**Table S6A**) including 248 luminal A, 134 luminal B, 132 basal, 71 ERBB2 and 17 normal-like BCs. A total of 517 genes exhibited more than 5 mutations in the 602 tumors. For each of them, the frequency of mutation was established and we identified gene mutations associated with a specific subtype by comparing the number of mutations with those observed in the others (Fisher, FDR<0.25 with odds ratio>1 dark grey colored in **Table S6D**). The first 15 most frequently mutated genes are reported in **Table 2** for each molecular subtype. *KCNB2* gene mutations were associated with luminal B tumors (Fisher, FDR<0.25 with odds ratio>1).

**Table 2:** NGS meta-analysis: first 15 most frequently mutated genes in each molecular subtype.

Basal		ERBB2		Luminal A		Luminal B	
(N = 132)		(N = 71)		(N = 248)		(N = 134)	
mutated genes	frequency (%)	mutated genes	frequency (%)	mutated genes	frequency (%)	mutated genes	frequency (%)
***TP53*** *	71%	***TP53*** *	68%	***PIK3CA*** *	43%	*PIK3CA*	31%
*TTN*	19%	*PIK3CA*	37%	***MAP3K1*** *	13%	*TP53*	29%
***USH2A*** *	11%	*MUC16*	14%	*GATA3*	13%	*GATA3*	13%
*FLG*	7%	***LRP1*** *	8%	*TP53*	11%	*TTN*	12%
*MUC16*	7%	***ERBB3*** *	8%	*TTN*	9%	*RYR2*	7%
*PIK3CA*	7%	***DNAH11*** *	8%	*CDH1*	9%	*RELN*	5%
*MUC17*	6%	*LRP2*	8%	*MLL3*	7%	*FAT3*	5%
***DNAH7*** *	5%	*TTN*	8%	*MAP2K4*	6%	*MLL3*	5%
*FAT3*	5%	***ATP1A4*** *	7%	*NCOR1*	5%	*MUC16*	5%
*SYNE1*	5%	***KIAA1109*** *	7%	*AKT1*	4%	***KCNB2*** *	4%
*DST*	5%	*CACNA1E*	7%	*RUNX1*	4%	*CENPE*	4%
*F5*	5%	*FLG*	7%	*PTEN*	4%	*DMD*	4%
*COL12A1*	5%	*MLL3*	7%	*MUC16*	4%	*MUC17*	4%
*COL6A3*	5%	***ASPM*** *	6%	*CTCF*	4%	*CDH1*	4%
*APOB*	5%	***DCC*** *	6%	*NEB*	4%	*MAP3K1*	4%

Gene mutation associated with the corresponding molecular subtype.

In an NGS subset [29], *TP53* mutations were enriched in luminal B tumors (p = 0.04) and in high grade tumors (p = 0.02). In our meta-analysis of all NGS samples, *TP53* mutations were enriched in only basal (FDR = 4×10^−18^) and ERBB2 (FDR = 5×10^−6^) tumors. The proportion of analyzed tumors and their heterogeneities could influence the results.

To identify co-occurring and mutually exclusive subtype-specific gene mutations, we retained in the 602 samples, the genes targeted by more than 3 mutations in each subtype. From the 103, 76, 98, 31 retained genes, we identified 74, 91, 121 and 52 co-occurring mutations, and 3 (*AKT1/PIK3CA*, *MAP3K1/TP53*, *MAP3K1/TTN*), 1 (*GATA3/PIK3CA*), 0 and 0 mutually exclusive gene mutations (p<0.05) in luminal A, luminal B, basal and ERBB2 tumors, respectively (**Tables S6E–S6H**, respectively).

To identify key luminal B genes that could be altered by several mechanisms, we crossed in **Table S6I** information between genes found in the meta-analysis as mutated in luminal B cases and those identified in our study as significantly altered in luminal B tumors (**Tables S2A–S2F**) or as potential luminal B candidates (**Table S3G**). *KCNB2* mutations were associated with luminal B tumors (Fisher, FDR<0.25 with odds ratio>1), and the gene was more frequently gained in luminal B than in the other subtypes (Fisher's exact test; FDR<0.05) (**Tables S2A–S2F**). Among luminal B candidate genes, only *CIT* (12q24) (3%), *CHD6* (20q12) (2%), *MDN1* (6q15), *SRGAP1* (12q14.2) (1.5%), *UTRN* (6q24), *BRCA1* (17q21) and *EVPL* (17q25) (less than 1%) were also targeted by mutations.

## Discussion

Luminal B breast tumors have aggressive clinical and biological features [32]. A better definition of molecular alterations could improve our understanding of this tumor phenotype and allow the identification of new diagnostic and/or therapeutic targets. Here, we have first established and compared the genomic and gene expression profiles of 32 luminal B tumors and 156 tumors from other subtypes to constitute a repertoire of luminal B candidate genes. Second, in a subset of 109 breast tumors, we have compared the different methylated DNA landscapes associated with each subtype. Third, we have surveyed molecular alterations in some cancer genes and compared the results with a meta-analysis done with NGS data on 875 BCs.

This study depicts for the first time a luminal B genomic and epigenomic landscape including frequent *FOXO3, PIK3CA* and *TP53* mutation, with significant *ZNF703*, *MYC*, *CCND1*, *ZNF217* gains/amplifications, *FOXO3*, *CDKN2A*, *L3MBTL4*, various 6q regions, *ARID1B* losses and *FOXO3* mutations. In this luminal B context, (i) 237 and 101 luminal B-specific candidate oncogenes and TSGs presented a deregulated expression in relation with their CNAs; as well as (ii) low *ASS1*, *C6ORF145/PXDC1*, *ZFP36L2* and high *C3ORF67, C12ORF60, H2AFJ, RAB11FIP1* gene expression in relation with their DNA methylation levels. From the meta-analysis, only *KCNB2* gene mutation was associated with this subtype. Moreover, in a very large tumor set, we confirmed the luminal B specificity of 100 candidate oncogenes and showed for the first time that the overexpression of 61 of them are pertinent predictor of poorer patient outcome in luminal breast carcinomas independently of PR status. Compared to previous studies, our analysis point to 6q deletions and *FOXO3* mutations as important actors of luminal B genesis.

### Specific gains and amplifications target oncogene candidates in luminal B tumors

The high proportion of complex “sawtooth and firestorm” genomic profiles observed in luminal B tumors suggests a high level of genomic instability. Expectedly, among the most common specific alterations in luminal B tumors (≥30%) were the amplified 8p11-p12 and 11q13.1-q13 regions. In these regions *ZNF703*, *FGFR1* and *CCND1* have already been found associated with luminal B BCs [9], [19], [46], [51]. The comparison between luminal B and luminal A genomic profiles showed only differences in the frequency of these, which may contribute to phenotypic differences. ZNF703 interacts with WDR68/DCAF7, PHB2 and HSP60 and induces transcriptional repression [19]. Here, we found that *WDR68/DCAF7* (17q23.3) is indeed a candidate luminal B oncogene. Genes of the 8p11 region were coamplified with *ZNF703*, supporting the idea that the 8p amplicon carries multiple genes, such as *RAB11FIP1/RCP* [47], *FGFR1* [54] and *PPAPDC1B* [45], which contribute to the luminal B phenotype.

In the 8p12/11q13 coamplified regions [55], *EIF4EBP1* and *RPS6KB2* could be co-targeted and play a synergistic role associated with the development and cancer progression as AKT/MTOR activators [56]. The recurrent 8p12/11q13 co-amplification was often accompanied by gain of 17q25.1 containing *RPS6KB1*, a paralog of *RPS6KB2*, suggesting again the involvement of the AKT/MTOR pathway in luminal B oncogenesis.

### Specific losses target TSG candidates in luminal B tumors

Chromosome arm 6q showed frequent deletions in luminal B tumors. Several 6q regions were lost and rare homozygous or focal deletions of *ARID1B*, *ASCC3*, *FOXO3, PARK2*, *MLLT4/Afadin* and *UFL1/NLBP* genes were observed. Deletion of 6q is also frequent in a variety of cancers [57]. Several other chromosomal regions were targeted by losses suggesting the existence of several potential TSGs in luminal B cancers (**Table S7**). We identified luminal B candidate TSGs *PNRC1* (6q15), *PTPRK* (6q22.33) and *L3MBTL4* (18p11.31). We previously reported *L3MBTL4* as a potential BC TSG. PNRC1 is a coactivator of nuclear receptors such as ER. PTPRK negatively regulates the transcriptional activity of ß-catenin in cancer cells.

### Gene expression deregulation of luminal B candidate genes could perturb epigenetic regulation

Eleven of the luminal B candidate genes we found have been associated with endocrine resistance [33], [34], [44]. Upregulation of *FOXM1* [58] could explain in part the DNA methylated landscape characterizing luminal B tumors. The deregulation of *ARID1B, ASHL2*, *CHD6, KDM4C/JMJD2C, L3MBTL4* and *ZFP161* suggests an important perturbation of the epigenetic regulation in luminal B tumors.

BC subtypes have specific methylation profiles [20], [21]. Luminal B and basal BCs were reported as the most and least frequently methylated, respectively [21]. Our data are in agreement with these observations; the median methylation level of the 4,545 subtype-associated promoters was the highest in the luminal and ERRB2 subtypes and the lowest in the basal subtype. High DNA methylation level associated with the luminal B subtype targeted *CITED4*, *SP100*, *SAMD9L*, *DCR1*, *FBXO32*, *ASS1*, *FAM78A* and *STAT5A* genes previously reported as TSGs or associated with tumor progression (**Table S7**). None of them is located in 6q. The two specific luminal B candidates, *ASS1* and *ZFP36L2* downregulated in relation with DNA methylation have been reported targeted in cancers. DNA methylation of the *ASS1* promoter leading to its downregulation was associated with poor prognosis and chemoresistance in various cancers. Several phase I/II clinical trials of the arginine-lowering drug, pegylated arginine deiminase, showed encouraging evidence of clinical benefit and low toxicity in patients with ASS1-negative tumors that might be extended to luminal B tumors.

Finally, we further noted that multiple alternatively spliced transcript variants have been described for candidate genes *ASB13, CIT, CPSF1, EPN2, LSM1, PDCD4, RNF146, TAF4, VTCN1*. Transcripts of *CIT*, *CPSF1, PDCD4* and *TAF4* are overlapped by MIR1178, MIR939 and MIR1234 (both), MIR4680 and MIR1257, respectively [59]. Changes in MIR expression may also contribute to luminal B tumorigenesis by modulating the functional expression of critical genes involved in cancer growth and metastasis.

### Mutation status

As expected, *TP53* and *PIK3CA* were among the most frequently mutated genes in our luminal B series. *FOXO3* and *RUNX3* genes were lost and mutated. A possible role for RUNX3 as a tumor suppressor in ER+ BCs has been suggested [60], [61]. FOXO3 functions as a tumor suppressor in both ER+ and ER− BCs [62], [63]. Its nuclear localization and subsequent transcriptional activity is a marker of good prognosis among breast cancer patients [64].

Our meta-analysis of NGS samples showed the frequent mutations of *PIK3CA*, *TP53* and *GATA3* in luminal B tumors but also that *KCNB2* gene mutations were, for an unexplained reason, associated with this aggressive subtype. A total of 91 co-occurring mutations and *GATA3/PIK3CA* gene mutual exclusive mutations completed the landscape associated with luminal B tumors. Surprisingly, *RUNX3* and *FOXO3* mutations were not found in the NGS studies. This may be due the limited coverage and low depth of these early analyses, or to BC heterogeneity and the large number of alterations that could lead to similar deregulations of particular pathways.

### Potential targeted therapy in luminal B breast cancer

Hormonal therapy is the preferred treatment for about two-thirds of all BC patients whose tumor expresses ER. ER+ BCs are commonly treated with anti-estrogens or aromatase inhibitors [65]. Luminal B tumors respond less than luminal A tumors to such treatments. Seven of the luminal B candidate oncogenes we have identified could be targeted [43]. Several phase I/II clinical trials targeting IGF, FGF and PI3K/AKT pathways in luminal B BC have been reported [32]. Except for the IGF pathway, our data are in agreement with the potential activation of the FGF, PI3K/AKT, PIK3/MTOR pathways subsequent to the overexpression of candidate oncogenes such as *RPS6KB1*, *RPS6KB2*, *EIF4EBP1*, *FGFR1*, *FRS2*, and *RAB11FIP1* and the high frequency of *PIK3CA* mutations. Interestingly, targeting the PI3K/AKT/mTOR pathway is one of the most-promising therapeutic approaches to reversing endocrine resistance for ER positive breast cancer (for review, [66]). Therapeutic strategies combining endocrine treatment and signaling pathways inhibition (such as targeting growth factor receptors (ERBB2) PI3K/AKT/mTOR, CDK4 and CDK6, MDM2–TP53 interaction, FGFR pathway aberrations and histone deacetylases) might be pertinent [66]. However, the luminal B molecular heterogeneity depicted by its complex genomic and epigenomic landscape strongly suggests that other potential targets could also exist and should be identified for each patient. The candidate oncogene *RECQL4*, which might be involved in endocrine resistance [44], could be also a potential therapeutic target [43] as recently shown in breast cancer cells [52].

The targeting of candidates such as YWHAZ and its coregulated proteins such as FOXM1, or NHERF1, could also restore endocrine sensitivity and reduce the risk of BC recurrence [32], [34], [44], [67], [68]. FOXM1 has a significant role in endocrine therapy resistance [67]. In endocrine therapy-resistant BCs, high FOXM1 expression could result from the loss of FOXO function as transcriptional repressor.

In conclusion, this refined molecular dissection of luminal B BCs has pointed to both new and well-known specific candidates. Some code for proteins that participate to the same signaling pathways including those known to cross-talk with ER signaling pathways. Many of the candidate genes have not been previously reported in breast cancer, and deserve further functional validation. Similarly, further characterization of the 6q TSGs is an important goal. This should help better understand pathways and mechanisms affected, and find new therapeutic targets.

## Supporting Information

Figure S1 Whole-genome expression profiling of 188BCs. **A**) Hierarchical clustering of 188 samples and 13,031 probe sets with significant variation (sd>0.5) in mRNA expression level across the samples. Each row of the data matrix represents a gene and each column represents a sample. Expression levels are depicted according to the color scale shown at the bottom. Red and green indicate expression levels respectively above and below the median. The magnitude of deviation from the median is represented by the color saturation. The dendrogram of samples (above matrixes) represents overall similarities in gene expression profiles and is zoomed in B. Colored bars to the right indicate the locations of 6 gene clusters of interest (ECM means extra-cellular matrix). **B**) Dendrograms of samples. *Top*, four groups of tumor samples (designated I to IV) are evidenced and delimited by orange vertical lines. Below the dendrogram, are some histoclinical and molecular features of the samples: from top to bottom, intrinsic molecular subtypes (dark blue for luminal A, light blue for luminal B, red for basal, pink for ERBB2-overexpressing, and green for normal-like)^4^, GC (genome complexity) (green for simplex, orange for complex saw tooth and brown for complex firestorm), SBR grade (white for grade I, grey for II, and black for III), and IHC ER, ERBB2, and P53 status (white for negative, and black for positive). Crossed white boxes mean not assigned samples.
(TIFF)

Figure S2 Integrated comparative analysis of luminal B *vs* non luminal B BCs. The depicted pipeline integrates genomic and gene expression data analyses. The three successive steps are numbered 1, 2 and 3.
(TIF)

Figure S3 Integrated comparative DNA methylation and gene expression analysis associated with breast cancer molecular subtypes. The depicted pipeline integrates DNA methylation and gene expression data analyses.
(TIF)

Figure S4 Genomic characterization of 188 BCs in regard to their molecular subtype. CNA frequencies and the repartition of genomic patterns are described for each molecular subtype. Amplification, gains, homozygous and hemizygous losses CNAs are distinguished by different colors.
(TIFF)

Figure S5 Specific regions targeted by CNAs in luminal B BCs. Results of supervised analysis comparing CNA frequencies between luminal B and luminal A, luminal B and basal, luminal B and ERBB2 and luminal B and non-luminal B tumors. Only amplification, gains, homozygous and hemizygous losses associated with luminal B tumors are shown (Fisher's exact test; FDR<0.05) for each chromosome. They are distinguished by illustrated colors. The successive comparisons show distinct regional CNAs significantly associated with luminal B tumors compared to those found in each of the other major molecular subtypes.
(TIFF)

Figure S6 Examples of chromosome 6 aCGH profiles in luminal B tumors. Tumors exhibit various 6q regional losses as well as rare homozygous deletions and small deleted regions targeting *MLLT4*, *ARID1B*, *PARK2*, *FOXO3A, UFL1/NLBP, ASCC3* genes.
(TIFF)

Figure S7 Examples of chromosome 9 aCGH profiles in luminal B tumors. Tumors exhibit various 9p regional losses as well as rare focused deletions and short deleted regions. (**A**) Tumor T17253 on the left does not present any CNA. Tumors T13139, T11305, T13694, T15354, T13148, T10798, T13728, T2362, and T13469 exhibit various copy number losses along the short arm of chromosome 9 suggesting at least two common lost regions involved in the luminal B tumors (bold lines to the right). (**B**) The focused deletions observed in tumors T8189 and T6137-2 target the *MTAP-CDKN2B-CDKN2A* (9p21.3) and *BNC2* (9p23.2) genes, respectively. (**C**) The genomic profiles observed in tumors T2362, and T13469 show a short common deleted region spanning from centromere to telomere, *C9ORF93, PSIP1, C9ORF59, FREM1, CERF1, ZDHHC21* and *NFIB* genes.
(TIFF)

Figure S8 Correlation between gene expression and genome alterations on the 8p11.1-p12 and 11q13.1-q13.4 regions. Genomic and gene expression profiles were established for 188 breast tumors (32 luminal B and 156 non luminal B identified at the top by blue and grey boxes, respectively) in the 8p11.1-p12 (top), and 11q13.1-q13.4 (bottom) regions. For each region, heatmaps for genome copy number and gene expression profiles are consecutively drawn. Genome copy number was measured by aCGH on probes or groups of probes spanning each of these regions. Red indicates increased copy number and green indicates decreased copy number. In the heatmap tumors are organized from the tumor that presented the highest copy number gains and amplification to the tumor that exhibited the most copy number losses. The next heatmap was established with the expression of the independent genes located on the corresponding region and profiled in the same 188 tumors similarly organized. For gene copy number and gene expression heatmaps, we used colour scale limits from −3 to +3 and −2 to +2, respectively. Next to the right, are plotted genes successively selected by steps I, II and III of the integrated analysis “aCGH & mRNA expression” as defined by the work pipeline (Figure S2). Grey and red lines correspond to rejected and selected genes, respectively. Among genes with an expression level that varied according to CNAs, we retained genes showed significant differences (vertical line) in copy number gains correlated with upregulated expression in luminal B compared to non-luminal B tumors. They were qualified as potential oncogenes.. For each region, only the first five most significant are listed. *ZNF703* and *CCND1* genes were the most significant candidate oncogenes for the 8p11.1-p12 (top) and 11q13.1-q13.4 (bottom) regions, respectively.
(TIFF)

Figure S9 Luminal B candidates and gene CNAs landscape. The Circos diagram presents from outside to inside, luminal B candidate genes, luminal B altered chromosomes, luminal B regional CNAs colored in red and green for significant gains and losses, respectively. Oranges and grey arcs indicate respectively genes/regions that present significant mutually exclusive and co-occurring luminal B CNAs (FDR<0.05) as identified in **Table S3N**.
(TIF)

Figure S10 Primers design for EpiTyper analysis of *RASSF1* promoter gene. *RASSF1* primers were designed for EpiTYPER™ Mass-ARRAY® system approach (SEQUENOM®, USA), to compare *RASSF1* DNA methylation data obtained by two independent methods. (**A**) *RASSF1* DNA methylation profiles of 15 ER+ and 33 ER- breast tumors established with their median normalized M values obtained for each *RASSF1* oligonucleotide present on the human promoter array (Agilent Technologies). (**B**) For the detection and quantitative analysis of DNA methylation, the EpiTyper approach used eight amplicons spanning the chr3:50,349,000–50,352,780 region including the *RASSF1* promoter and was covered by nine *RASSF1* oligonucleotides in the promoter array.
(TIF)

Figure S11 Comparison of *RASSF1* DNA methylation data obtained by two independent methods. (**A**) Hierarchical clustering established for 48 breast tumors samples with the methylation data of the 95 CpG within the *RASSF1* promoter and measured by the by EpiTyper method. Each row of the data matrix represents a CpG and each column represents a sample. DNA methylation variations are depicted according to the color scale shown at the bottom. Red indicates increased DNA methylation level and green indicates decreased DNA methylation level. The dendrogram (above matrixes) of samples represents overall similarities in DNA methylation profiles and is zoomed in the upper right part. The hierarchical clustering distinguished ER+ and ER− tumors (Fisher, p = 4.3 10^−3^). (**B**) For each sample, median methylation ratios (EpiTYPER) were calculated, with four informative amplicons overlapping oligoprobes (SQ00001_RASF1_07, SQ00001_RASF1_12, SQ00001_RASF1_25, .SQ00001_RASF1_32 mentionned by arrows) used to calculate Methylation Score. We observed a strong correlation between median methylation ratios and methylation score (Pearson correlation = 0.66, p = 4.6 10^−7^) calculated from data established by EpiTYPER and promoter array approaches, respectively.
(TIF)

Figure S12 Genes exhibiting a molecular subtype specific deregulated expression in relation with a molecular subtype specific methylation level variation. For luminal B (**A**), luminal A (**B**), ERBB2 (**C**) and basal (**D1–D4**) molecular subtypes are represented genes exhibiting a significant (i) DNA methylation level variation of their gene promoter (ANOVA, upper part) and (ii) gene expression deregulation compared to the other molecular subtypes (ANOVA and p-value showing significant difference, lower part).
(TIF)

Figure S13 Examples of mutation of *RUNX1* in breast cancer. **a**. Sequence profile of the mutated *RUNX1* allele, demonstrating base change in the forward sequence at the position indicated by an arrow. The corresponding sequence is shown above. **b**. Genomic organization of *RUNX1* gene and RUNX1 protein. Located at 21q22.12 chromosomal band, the *RUNX1* gene spans the chr21:36,160,098–36,421,595 region. The gene map established within Mb scale was extracted from the build GRCh37/hg19 from NCBI (February 2009 version) while its sequence (Ensembl Transcript ID ENST00000300305) was extracted from Ensembl database (http://www.ensembl.org/Homo_sapiens/), which is based on the Ensembl release 48 - Dec 2007 assembly of the human genome. Functional (i.e. RUNT and RUNXI [for RUNX Inhibitor domain], as defined by PFAM accession numbers PF00853 and PF08504, respectively) and motifs of the RUNX1 protein were positioned according to the SMART program (http://smart.embl-heidelberg.de/). Nucleotide (cDNA level) and deduced aminoacid sequences of the RUNX1 protein are positioned above and below the corresponding protein, respectively. **c**. The mutations observed in tumor samples are located with respect to the modified aminoacid of the RUNX1 protein.
(TIF)

Figure S14 Examples of mutation of *FOXO3* in breast cancer. From top to the bottom, in the 6q21 chromosomal band, the *FOXO3* gene spans the chr6:108,881,026–109,005,971 region. Exons 2 and 3 of *FOXO3* gene were analyzed for mutations. Sequence profile of the mutated *FOXO3* allele, demonstrating base change in the forward sequence at the position indicated by an arrow. The corresponding sequence is shown above.
(TIF)

Figure S15 Examples of aCGH profiles showing RUNX1 losses. (A) From left to right, aCGH profiles of chromosome 21 in cases T6744 (no apparent CNA), T9345, T8056, T50115, T9934, T11568, T15120, and T9207. Arrow shows *RUNX1* location on each genomic profile. Results show that *RUNX1* is targeted by potential breaks in T8056, T9207 and T15120, but also by regional deletions (samples T50115, T9934, T11568, T15120, and T9207). The genomic profiles of T11568 (**B**), T15120 (**C**), and T9207 (**D**) BCs presenting the smallest regional deletions were established with CGH analytics® software (Agilent Technologies), from centromere to telomere, within the genomic intervals [34.0–36.4 Mb] of the long arm of the chromosome 21. The common smallest deleted region observed in T9207 (**D**) involves *RUNX1*.
(TIF)

Results and References S1 In this section, results about: validation of our methylation approach; DNA methylation level associated with the other breast cancer molecular subtypes; and specific deregulated gene expression in relation with the DNA methylation level variation associated with the other breast cancer molecular subtypes; are presented with supplementary references.
(DOCX)

Table S1 **A**: Clinical and histological features of the 188 tumors. **B**: Description of the breast cancer data sets. **C**: FOXO3, MAP2K4, MAP3K1, PIK3CA, RUNX3 primers and PCR conditions used before sequencing analysis. **D**: Histoclinical correlations of the 188 BCs with the five SSP groups.
(XLSX)

Table S2 **A**: Comparison of CNA frequencies between luminal B and luminal A BCs. **B**: Identification of luminal B specific regions significantly targeted by CNAs after comparison of CNA frequencies between luminal B and luminal A BCs. **C**: Comparison of CNA frequencies between luminal B and basal BCs. **D**: Identification of luminal B specific regions significantly targeted by CNA after comparison of CNA frequencies between luminal B and basal BCs. **E**: Comparison of CNA frequencies between luminal B and non-luminal B BCs. **F**: Identification of luminal B specific regions significantly targeted by CNAs after comparison of CNA frequencies between luminal B and non-luminal B BCs.
(XLSX)

Table S3 **A**: Comparison of expression levels according to CNAs (Mann-Whitney ; FDR<0.05) inluminal B vs luminal A BCs. **B**: Identification of specific luminal B candidate genes from comparison between luminal B and luminal A BCs. A comparison with TCGA data [28] was done. **C**: Comparison of expression levels according to CNAs (Mann-Whitney ; FDR<0.05) in luminal B vs basal BCs. **D**: Identification of specific luminal B candidate genes from comparison between luminal B and basal BCs. A comparison with TCGA data [28] was done. **E**: Comparison of expression levels according to CNAs (Mann-Whitney ; FDR<0.05) in luminal B vs non luminal B BCs. **F**: Identification of specific luminal B candidate genes from comparison between luminal B and non-luminal B BCs. A comparison with TCGA data [28] was done. **G**: Summary of specific luminal B candidate genes from different comparisons between luminal B and the other molecular subtypes. **H**: 34 genes are exclusively deregulated in the comparison luminal B vs luminal A. **I**: 47 genes are exclusively deregulated in the comparison luminal B vs basal. **J**: 2 genes are exclusively deregulated in the comparison luminal B vs normal-like. **K**: 101 candidate oncogenes in the luminal B core. **L**: DAVID ontology analysis on the 101 genes of the luminal B core. **M**: The overexpression of 67 “luminal B core” candidate oncogenes is associated with poor MFS. A data comparison was done with those obtained for tumors classified with PAM50 SSP [48]. **N**: Mutually exclusive and co-occurring luminal B CNAs.
(XLSX)

Table S4 **A**: Histoclinical characteristics of the 117 samples (109 BC+8 NB) distributed in the three clusters defined by the unsupervised DNA methylation analysis. **B**: Supervised DNA methylation analysis comparing ER+ and ER- BCs. **C**: DNA methylation data analysis – 4,545 gene promoters exhibited a DNA methylation level different in at least one molecular subtype. **D**: 459 genes exhibited mRNA expression deregulation in relation with DNA methylation level. **E**: Genes exhibiting a mRNA expression deregulation in relation with DNA methylation level in the various molecular subtypes. A comparison with TCGA data [28] was done. **F**: Molecular subtype specific candidates presenting mRNA expression deregulation in relation with CNA and with DNA promoter methylation aberrations.
(XLSX)

Table S5 **A**: Criteria for our selection of the 9 sequenced genes. **B**: Gene mutation analysis of *ARID1A*, *ASXL1*, *FOXO3*, *L3MBTL4*, *MAP2K4*, *PIK3CA*, *RUNX1*, *RUNX3* and *TP53* in a large set of BCs including our panel of 188 BCs. **C**: Frequency analysis of genomic alterations targeting specific regions or genes in the five molecular breast cancer subtypes.
(XLSX)

Table S6 **A**: References of the six recent NGS studies used in the meta-analysis (dataset Meta-analysis). **B**: Frequency of somatic mutations identified in the 875 NGS breast tumors. **C**: Co-occurring and mutually exclusive mutations in the 875 NGS BCs (without molecular subtype distinction). **D**: Frequency of somatic mutations and association with molecular subtypes. **E**: Co-occurring and mutually exclusive gene mutations associated with luminal A subtype. **F**: Co-occurring and mutually exclusive gene mutations associated with luminal B subtype. **G**: Co-occurring and mutually exclusive gene mutations associated with basal subtype. **H**: Co-occurring and mutually exclusive gene mutations associated with ERBB2 subtype. **I**: genes targeted by several alterations mechanisms in luminal B subtype.
(XLSX)

Table S7 Genes commented in discussion with supplemental references.
(XLSX)
