# Supplementary figures and images for: Candidate Luminal B Breast Cancer Genes Identified by Genome, Gene Expression and DNA Methylation Profiling
Source: PLoS One. 2014 Jan 9;9(1):e81843. doi: 10.1371/journal.pone.0081843 (PMC3886975; doi:10.1371/journal.pone.0081843)

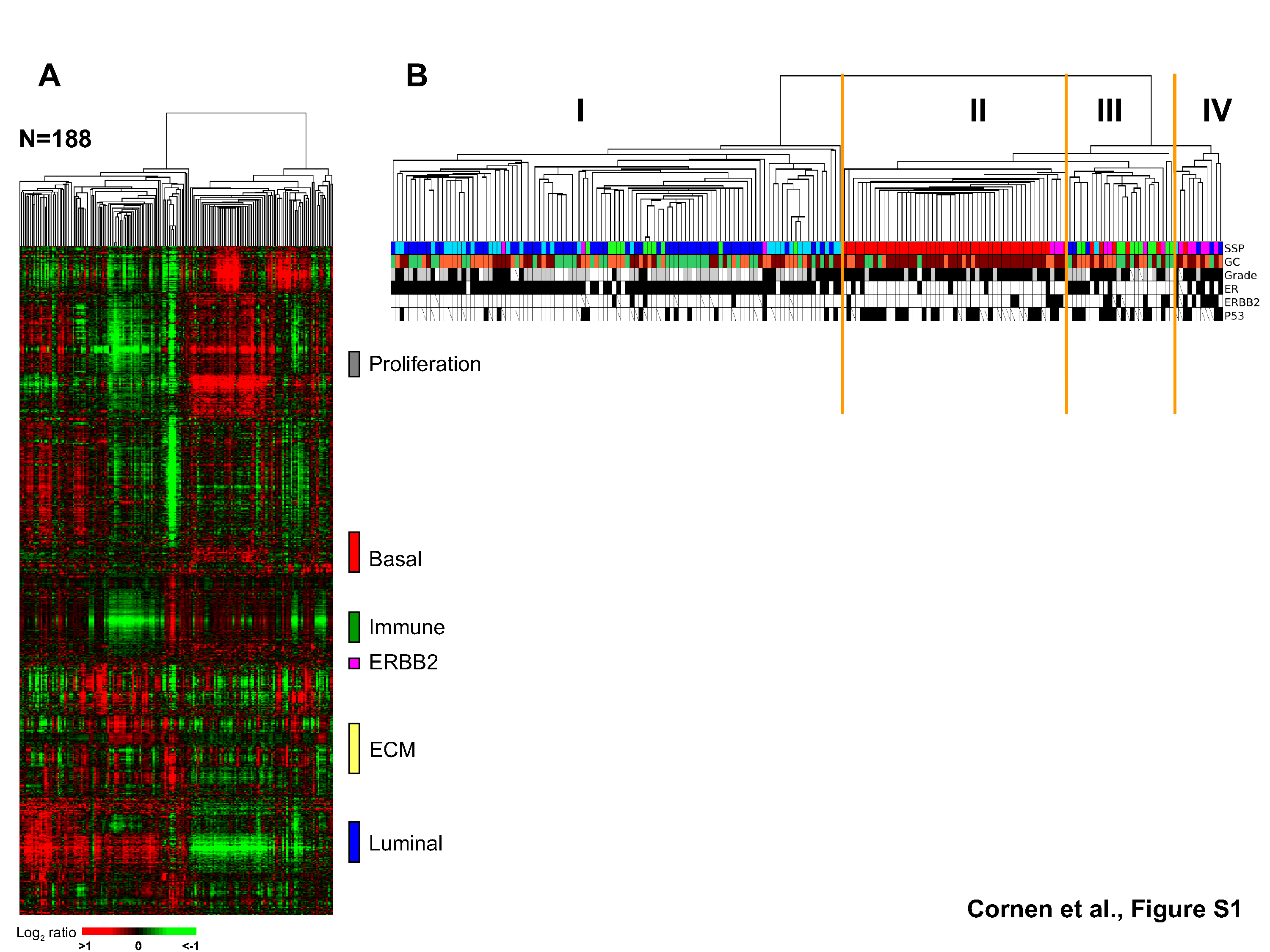

Supplement: Figure S1 — Whole-genome expression profiling of 188BCs. A) Hierarchical clustering of 188 samples and 13,031 probe sets with significant variation (sd>0.5) in mRNA expression level across the samples. Each row of the data matrix represents a gene and each column represents a sample. Expression levels are depicted according to the color scale shown at the bottom. Red and green indicate expression levels respectively above and below the median. The magnitude of deviation from the median is represented by the color saturation. The dendrogram of samples (above matrixes) represents overall similarities in gene expression profiles and is zoomed in B. Colored bars to the right indicate the locations of 6 gene clusters of interest (ECM means extra-cellular matrix). B) Dendrograms of samples. Top, four groups of tumor samples (designated I to IV) are evidenced and delimited by orange vertical lines. Below the dendrogram, are some histoclinical and molecular features of the samples: from top to bottom, intrinsic molecular subtypes (dark blue for luminal A, light blue for luminal B, red for basal, pink for ERBB2-overexpressing, and green for normal-like)4, GC (genome complexity) (green for simplex, orange for complex saw tooth and brown for complex firestorm), SBR grade (white for grade I, grey for II, and black for III), and IHC ER, ERBB2, and P53 status (white for negative, and black for positive). Crossed white boxes mean not assigned samples. (TIFF) [file pone.0081843.s001.tiff]

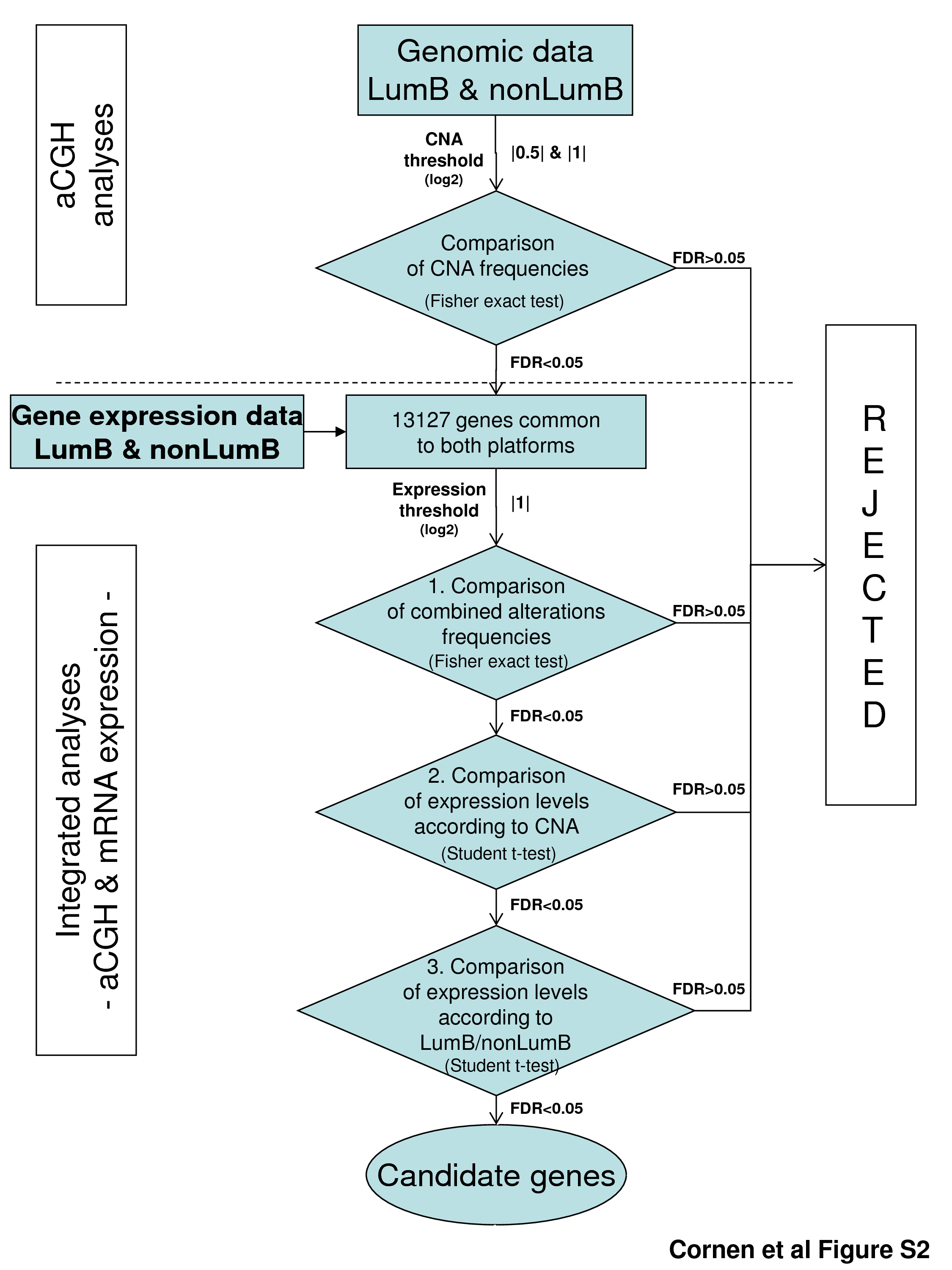

Supplement: Figure S2 — Integrated comparative analysis of luminal B vs non luminal B BCs. The depicted pipeline integrates genomic and gene expression data analyses. The three successive steps are numbered 1, 2 and 3. (TIF) [file pone.0081843.s002.tif]

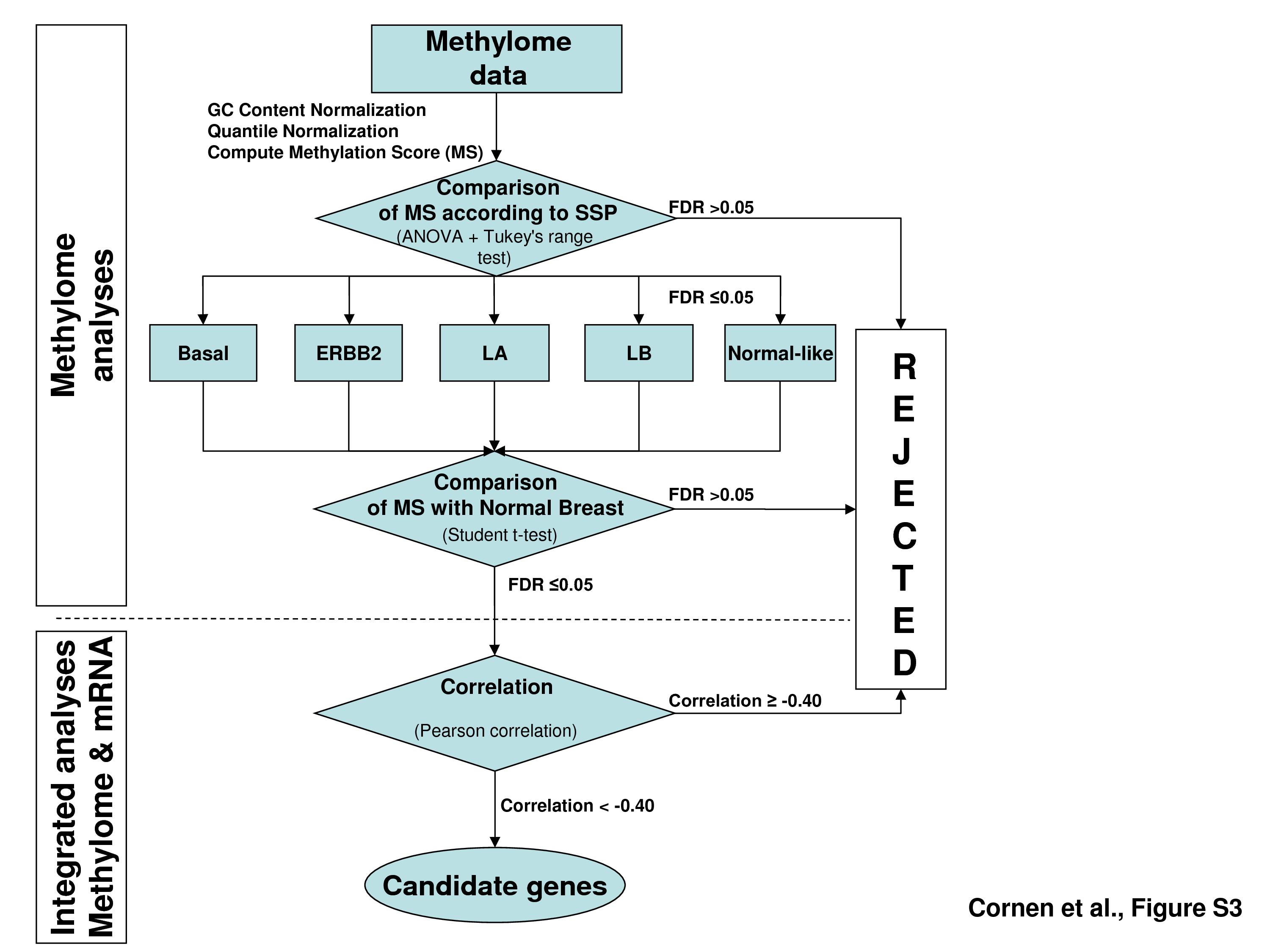

Supplement: Figure S3 — Integrated comparative DNA methylation and gene expression analysis associated with breast cancer molecular subtypes. The depicted pipeline integrates DNA methylation and gene expression data analyses. (TIF) [file pone.0081843.s003.tif]

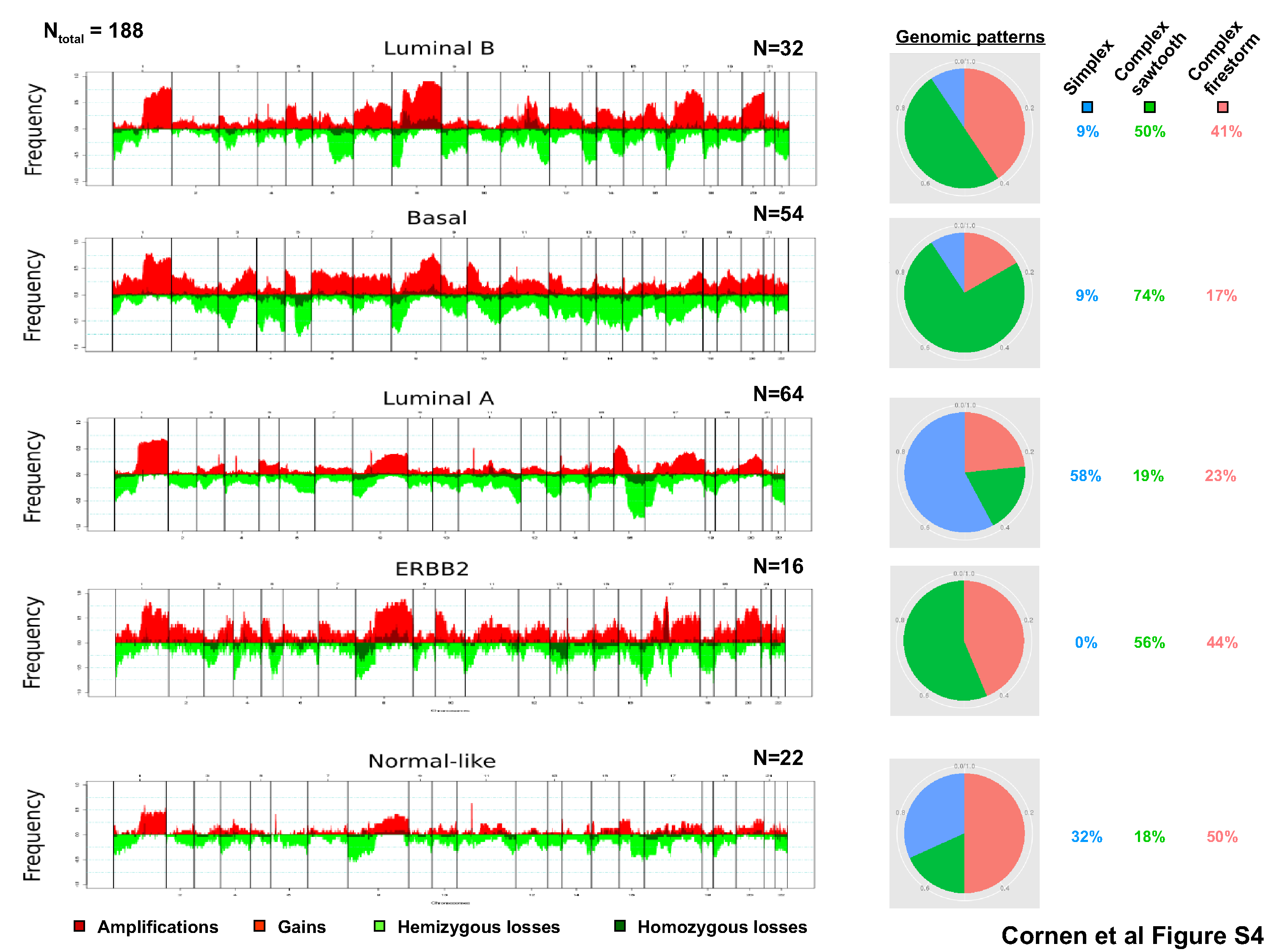

Supplement: Figure S4 — Genomic characterization of 188 BCs in regard to their molecular subtype. CNA frequencies and the repartition of genomic patterns are described for each molecular subtype. Amplification, gains, homozygous and hemizygous losses CNAs are distinguished by different colors. (TIFF) [file pone.0081843.s004.tiff]

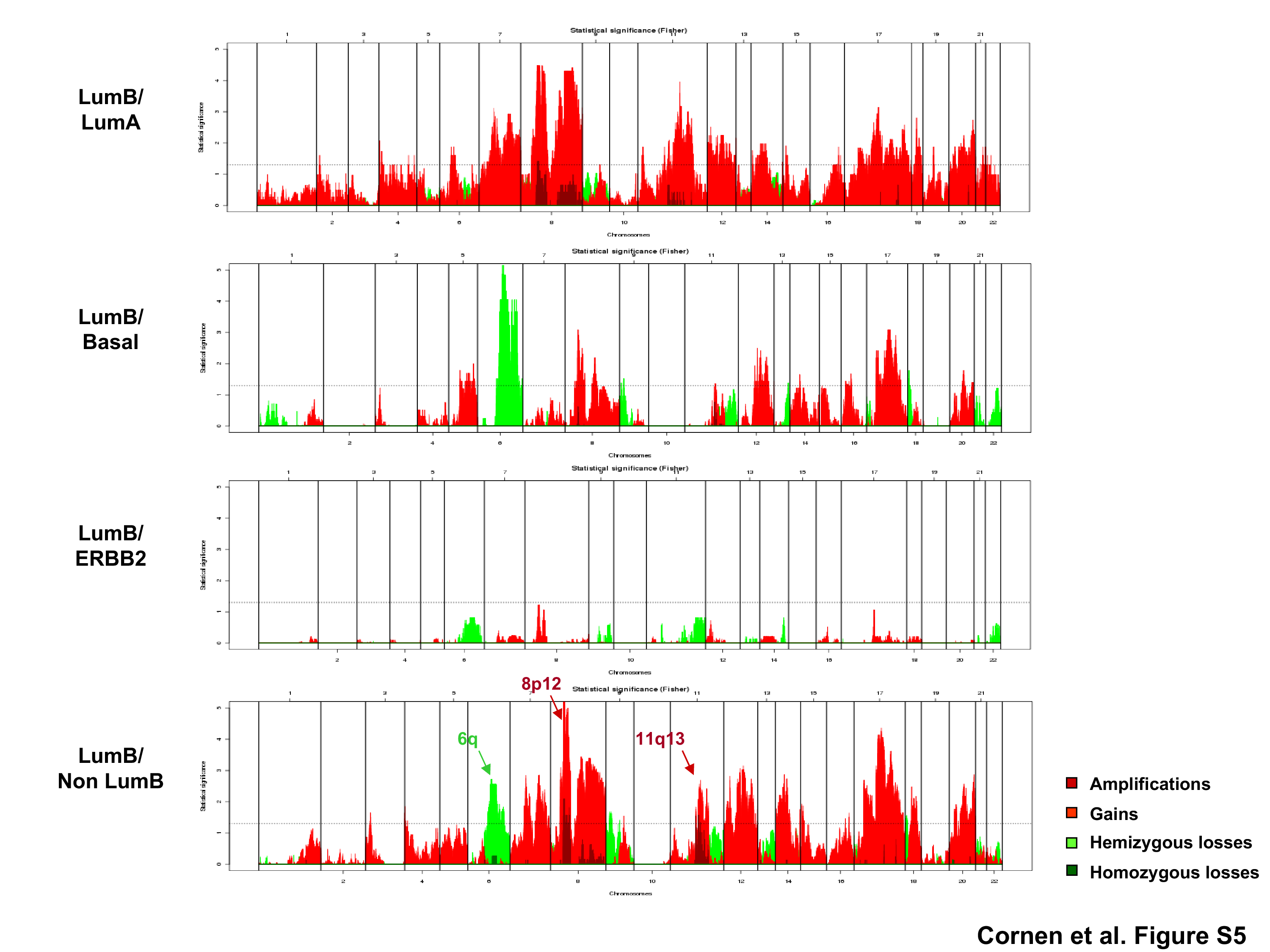

Supplement: Figure S5 — Specific regions targeted by CNAs in luminal B BCs. Results of supervised analysis comparing CNA frequencies between luminal B and luminal A, luminal B and basal, luminal B and ERBB2 and luminal B and non-luminal B tumors. Only amplification, gains, homozygous and hemizygous losses associated with luminal B tumors are shown (Fisher's exact test; FDR<0.05) for each chromosome. They are distinguished by illustrated colors. The successive comparisons show distinct regional CNAs significantly associated with luminal B tumors compared to those found in each of the other major molecular subtypes. (TIFF) [file pone.0081843.s005.tiff]

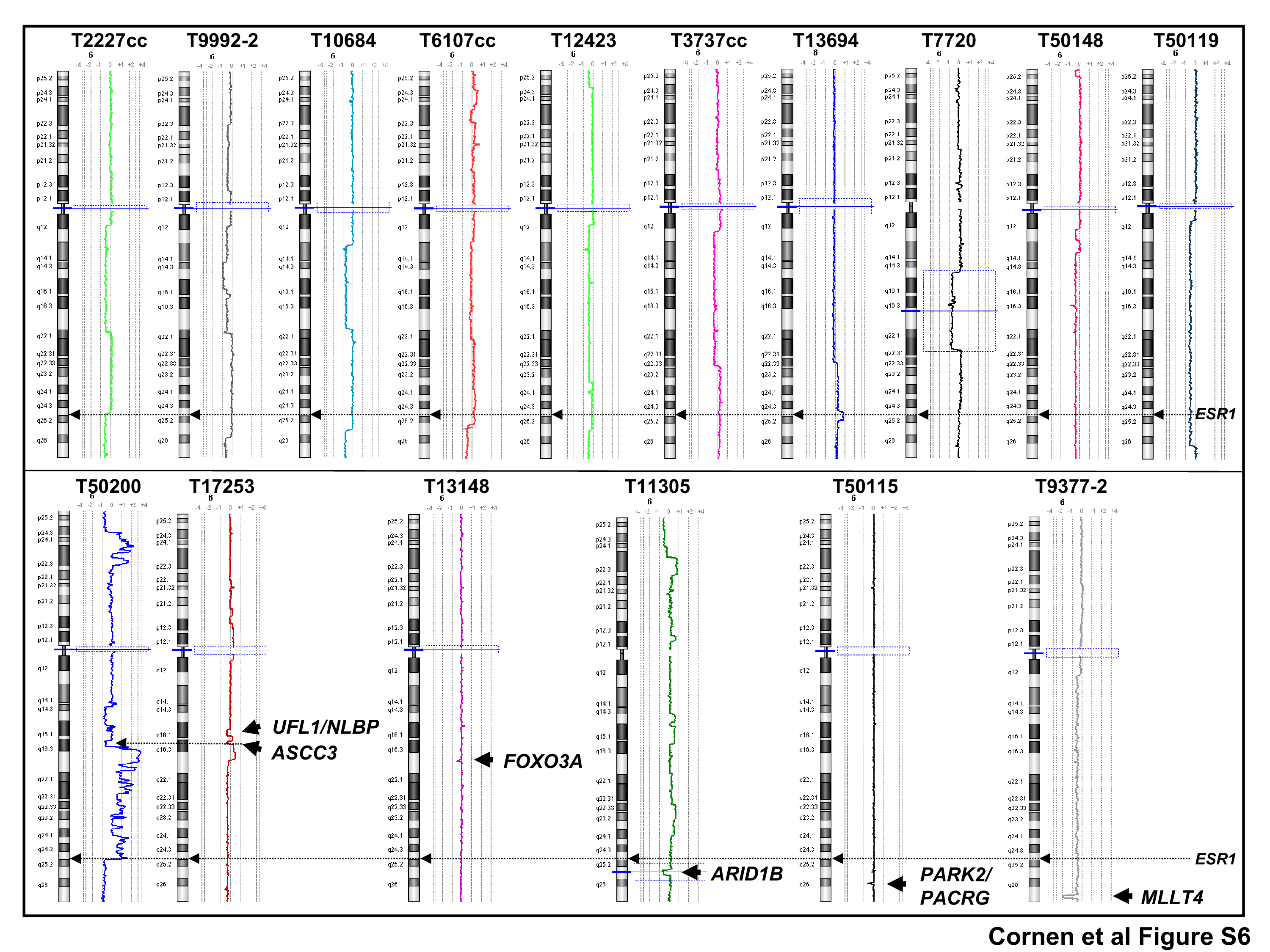

Supplement: Figure S6 — Examples of chromosome 6 aCGH profiles in luminal B tumors. Tumors exhibit various 6q regional losses as well as rare homozygous deletions and small deleted regions targeting MLLT4, ARID1B, PARK2, FOXO3A, UFL1/NLBP, ASCC3 genes. (TIFF) [file pone.0081843.s006.tiff]

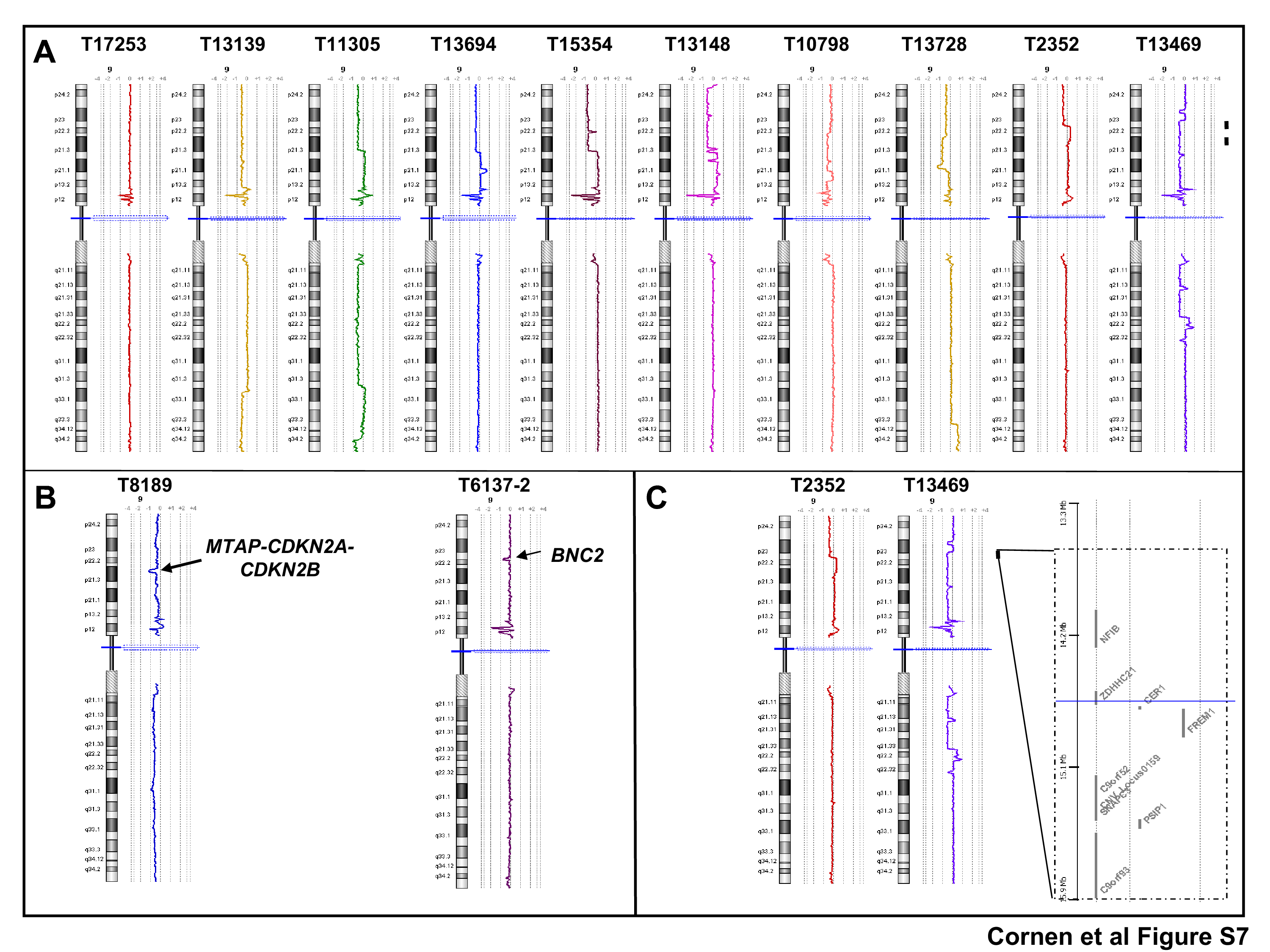

Supplement: Figure S7 — Examples of chromosome 9 aCGH profiles in luminal B tumors. Tumors exhibit various 9p regional losses as well as rare focused deletions and short deleted regions. (A) Tumor T17253 on the left does not present any CNA. Tumors T13139, T11305, T13694, T15354, T13148, T10798, T13728, T2362, and T13469 exhibit various copy number losses along the short arm of chromosome 9 suggesting at least two common lost regions involved in the luminal B tumors (bold lines to the right). (B) The focused deletions observed in tumors T8189 and T6137-2 target the MTAP-CDKN2B-CDKN2A (9p21.3) and BNC2 (9p23.2) genes, respectively. (C) The genomic profiles observed in tumors T2362, and T13469 show a short common deleted region spanning from centromere to telomere, C9ORF93, PSIP1, C9ORF59, FREM1, CERF1, ZDHHC21 and NFIB genes. (TIFF) [file pone.0081843.s007.tiff]

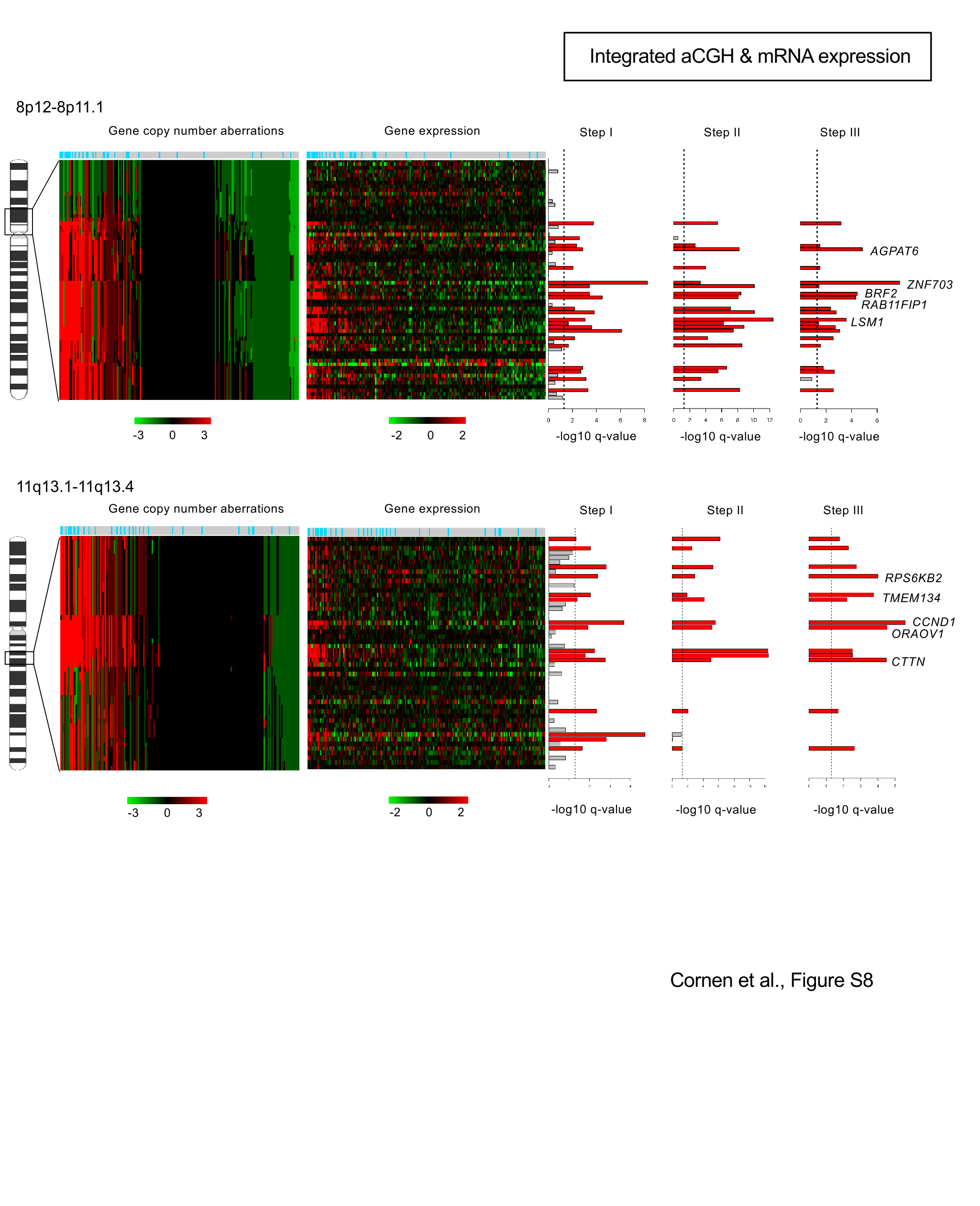

Supplement: Figure S8 — Correlation between gene expression and genome alterations on the 8p11.1-p12 and 11q13.1-q13.4 regions. Genomic and gene expression profiles were established for 188 breast tumors (32 luminal B and 156 non luminal B identified at the top by blue and grey boxes, respectively) in the 8p11.1-p12 (top), and 11q13.1-q13.4 (bottom) regions. For each region, heatmaps for genome copy number and gene expression profiles are consecutively drawn. Genome copy number was measured by aCGH on probes or groups of probes spanning each of these regions. Red indicates increased copy number and green indicates decreased copy number. In the heatmap tumors are organized from the tumor that presented the highest copy number gains and amplification to the tumor that exhibited the most copy number losses. The next heatmap was established with the expression of the independent genes located on the corresponding region and profiled in the same 188 tumors similarly organized. For gene copy number and gene expression heatmaps, we used colour scale limits from −3 to +3 and −2 to +2, respectively. Next to the right, are plotted genes successively selected by steps I, II and III of the integrated analysis “aCGH & mRNA expression” as defined by the work pipeline (Figure S2). Grey and red lines correspond to rejected and selected genes, respectively. Among genes with an expression level that varied according to CNAs, we retained genes showed significant differences (vertical line) in copy number gains correlated with upregulated expression in luminal B compared to non-luminal B tumors. They were qualified as potential oncogenes.. For each region, only the first five most significant are listed. ZNF703 and CCND1 genes were the most significant candidate oncogenes for the 8p11.1-p12 (top) and 11q13.1-q13.4 (bottom) regions, respectively. (TIFF) [file pone.0081843.s008.tiff]

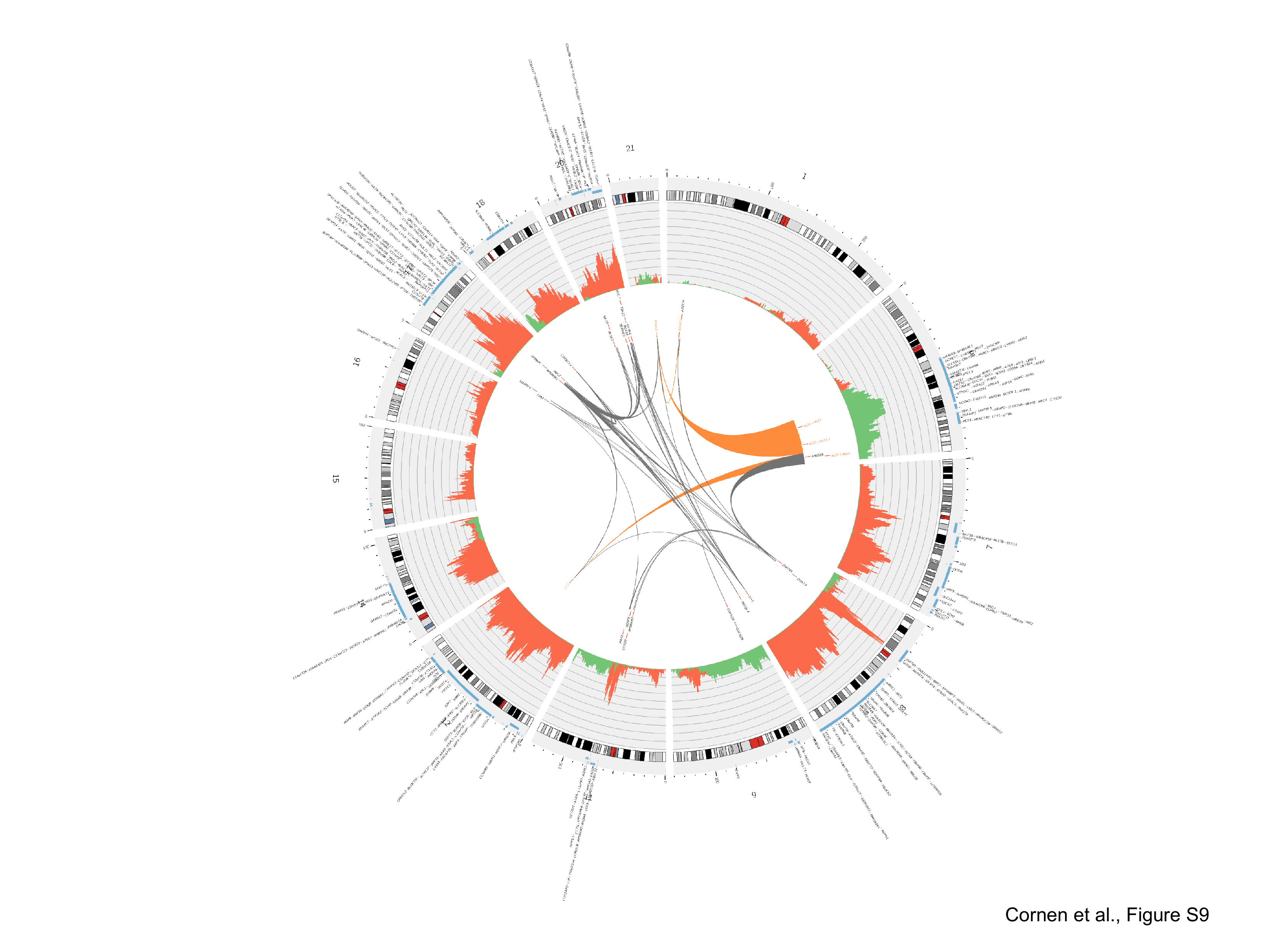

Supplement: Figure S9 — Luminal B candidates and gene CNAs landscape. The Circos diagram presents from outside to inside, luminal B candidate genes, luminal B altered chromosomes, luminal B regional CNAs colored in red and green for significant gains and losses, respectively. Oranges and grey arcs indicate respectively genes/regions that present significant mutually exclusive and co-occurring luminal B CNAs (FDR<0.05) as identified in Table S3N. (TIF) [file pone.0081843.s009.tif]

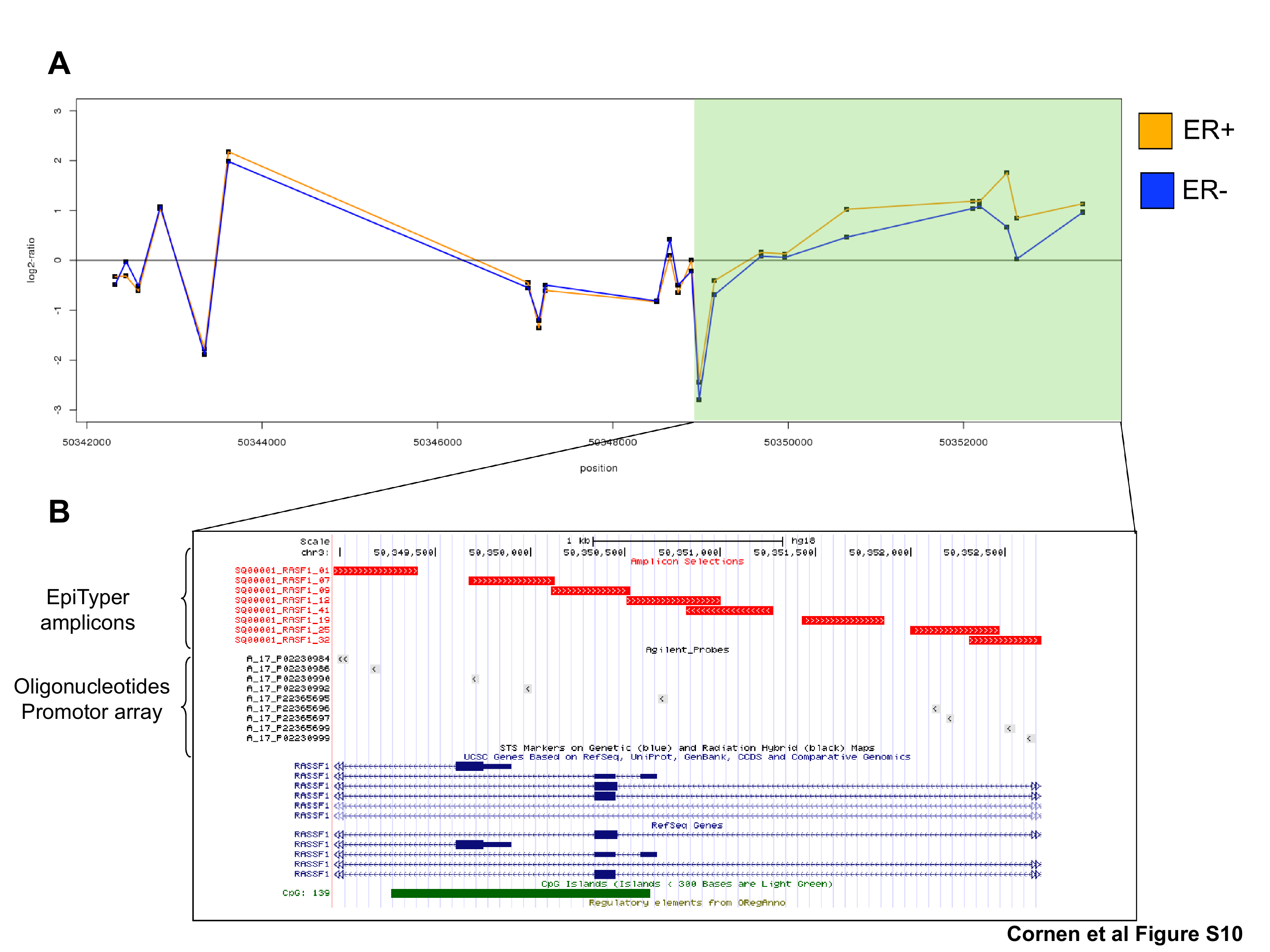

Supplement: Figure S10 — Primers design for EpiTyper analysis of RASSF1 promoter gene. RASSF1 primers were designed for EpiTYPER™ Mass-ARRAY® system approach (SEQUENOM®, USA), to compare RASSF1 DNA methylation data obtained by two independent methods. (A) RASSF1 DNA methylation profiles of 15 ER+ and 33 ER- breast tumors established with their median normalized M values obtained for each RASSF1 oligonucleotide present on the human promoter array (Agilent Technologies). (B) For the detection and quantitative analysis of DNA methylation, the EpiTyper approach used eight amplicons spanning the chr3:50,349,000–50,352,780 region including the RASSF1 promoter and was covered by nine RASSF1 oligonucleotides in the promoter array. (TIF) [file pone.0081843.s010.tif]

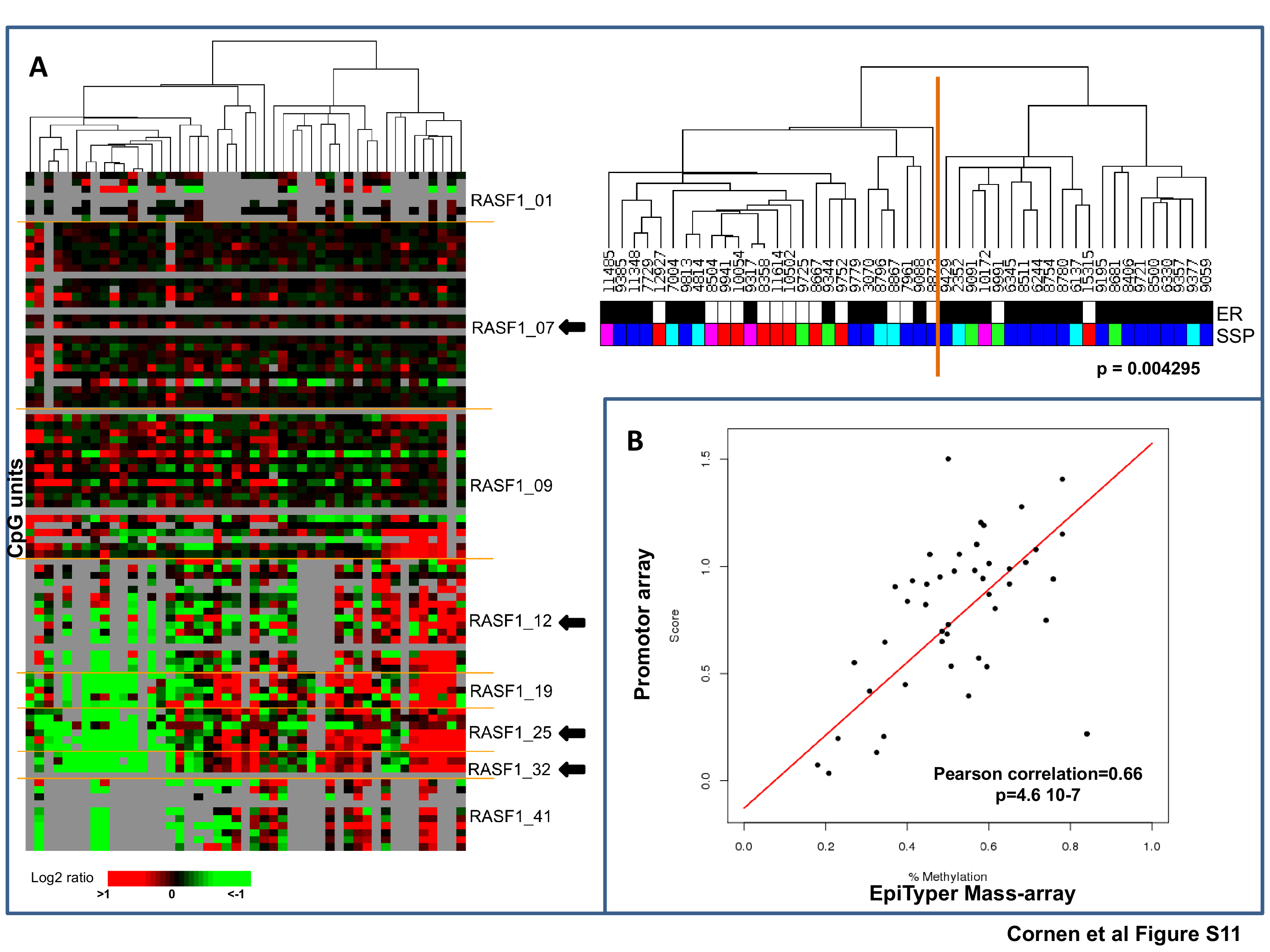

Supplement: Figure S11 — Comparison of RASSF1 DNA methylation data obtained by two independent methods. (A) Hierarchical clustering established for 48 breast tumors samples with the methylation data of the 95 CpG within the RASSF1 promoter and measured by the by EpiTyper method. Each row of the data matrix represents a CpG and each column represents a sample. DNA methylation variations are depicted according to the color scale shown at the bottom. Red indicates increased DNA methylation level and green indicates decreased DNA methylation level. The dendrogram (above matrixes) of samples represents overall similarities in DNA methylation profiles and is zoomed in the upper right part. The hierarchical clustering distinguished ER+ and ER− tumors (Fisher, p = 4.3 10−3). (B) For each sample, median methylation ratios (EpiTYPER) were calculated, with four informative amplicons overlapping oligoprobes (SQ00001_RASF1_07, SQ00001_RASF1_12, SQ00001_RASF1_25, .SQ00001_RASF1_32 mentionned by arrows) used to calculate Methylation Score. We observed a strong correlation between median methylation ratios and methylation score (Pearson correlation = 0.66, p = 4.6 10−7) calculated from data established by EpiTYPER and promoter array approaches, respectively. (TIF) [file pone.0081843.s011.tif]

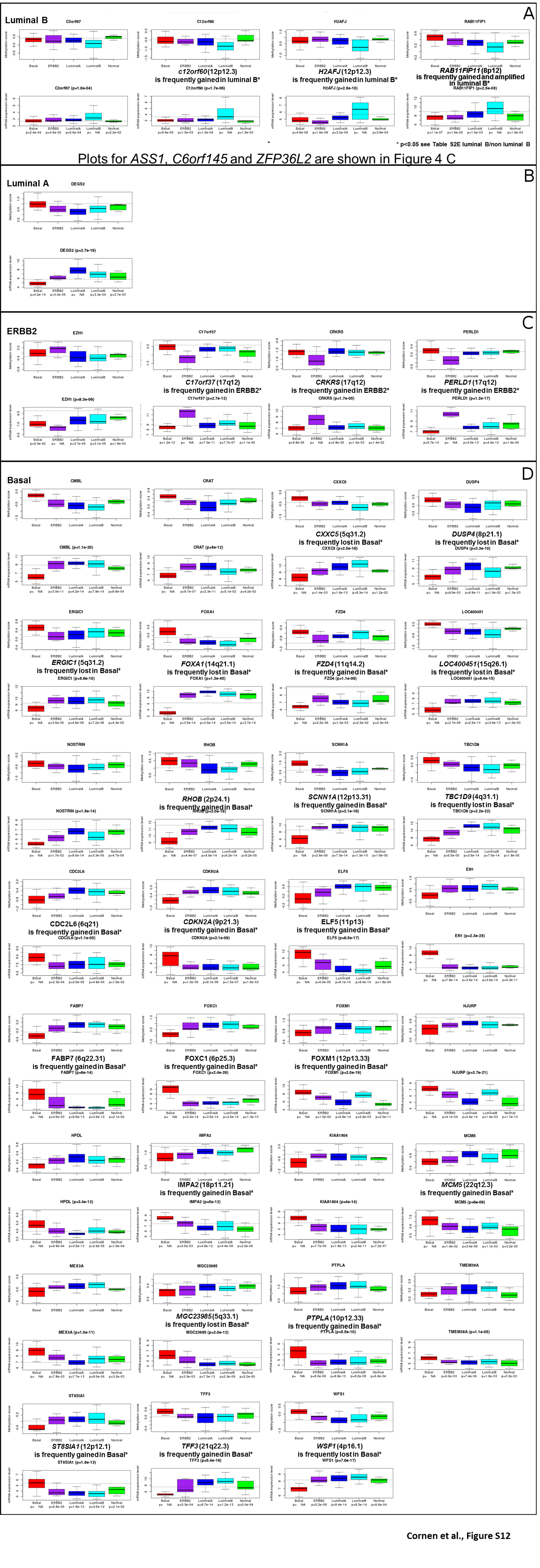

Supplement: Figure S12 — Genes exhibiting a molecular subtype specific deregulated expression in relation with a molecular subtype specific methylation level variation. For luminal B (A), luminal A (B), ERBB2 (C) and basal (D1–D4) molecular subtypes are represented genes exhibiting a significant (i) DNA methylation level variation of their gene promoter (ANOVA, upper part) and (ii) gene expression deregulation compared to the other molecular subtypes (ANOVA and p-value showing significant difference, lower part). (TIF) [file pone.0081843.s012.tif]

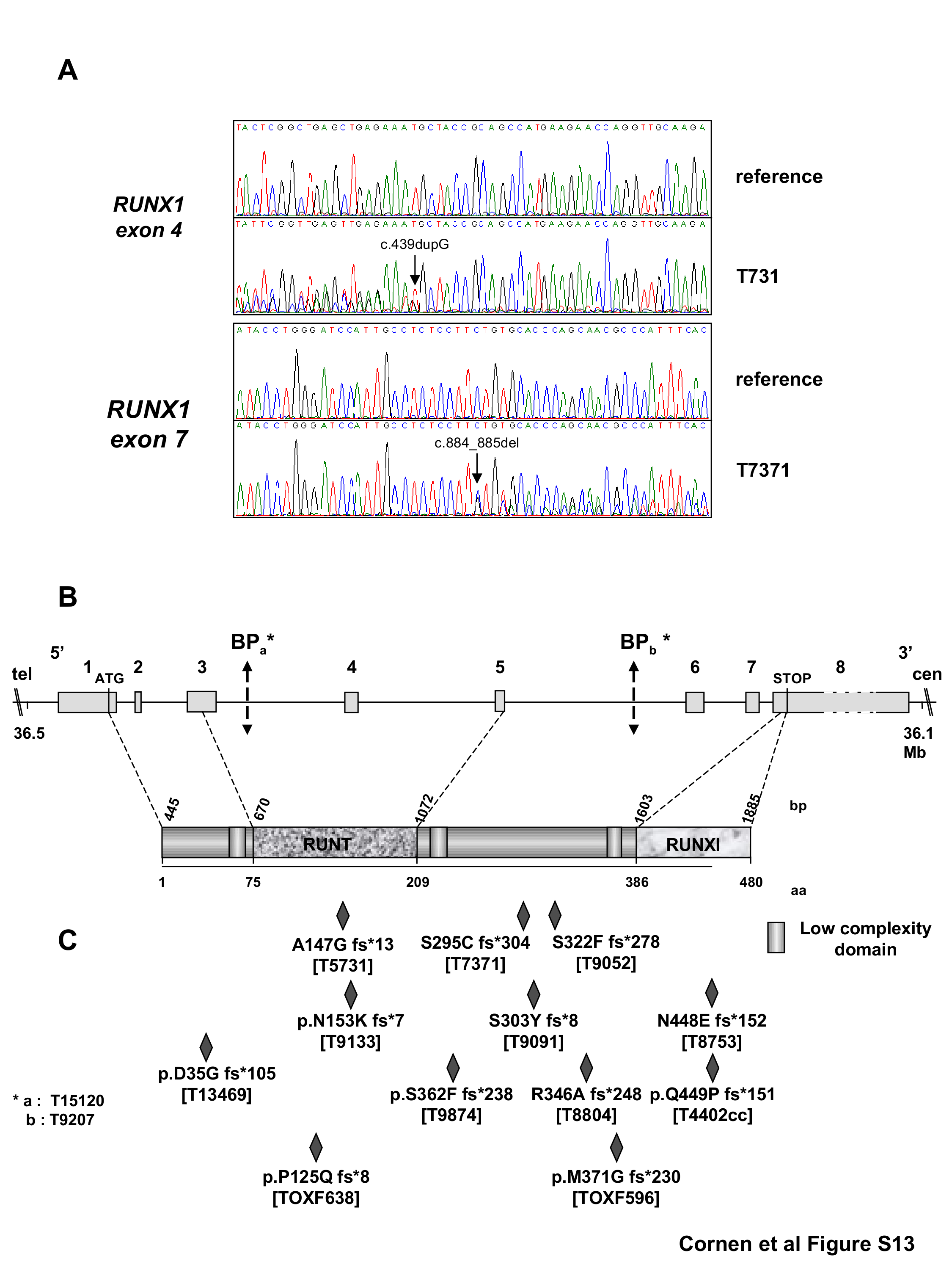

Supplement: Figure S13 — Examples of mutation of RUNX1 in breast cancer. a. Sequence profile of the mutated RUNX1 allele, demonstrating base change in the forward sequence at the position indicated by an arrow. The corresponding sequence is shown above. b. Genomic organization of RUNX1 gene and RUNX1 protein. Located at 21q22.12 chromosomal band, the RUNX1 gene spans the chr21:36,160,098–36,421,595 region. The gene map established within Mb scale was extracted from the build GRCh37/hg19 from NCBI (February 2009 version) while its sequence (Ensembl Transcript ID ENST00000300305) was extracted from Ensembl database (http://www.ensembl.org/Homo_sapiens/), which is based on the Ensembl release 48 - Dec 2007 assembly of the human genome. Functional (i.e. RUNT and RUNXI [for RUNX Inhibitor domain], as defined by PFAM accession numbers PF00853 and PF08504, respectively) and motifs of the RUNX1 protein were positioned according to the SMART program (http://smart.embl-heidelberg.de/). Nucleotide (cDNA level) and deduced aminoacid sequences of the RUNX1 protein are positioned above and below the corresponding protein, respectively. c. The mutations observed in tumor samples are located with respect to the modified aminoacid of the RUNX1 protein. (TIF) [file pone.0081843.s013.tif]

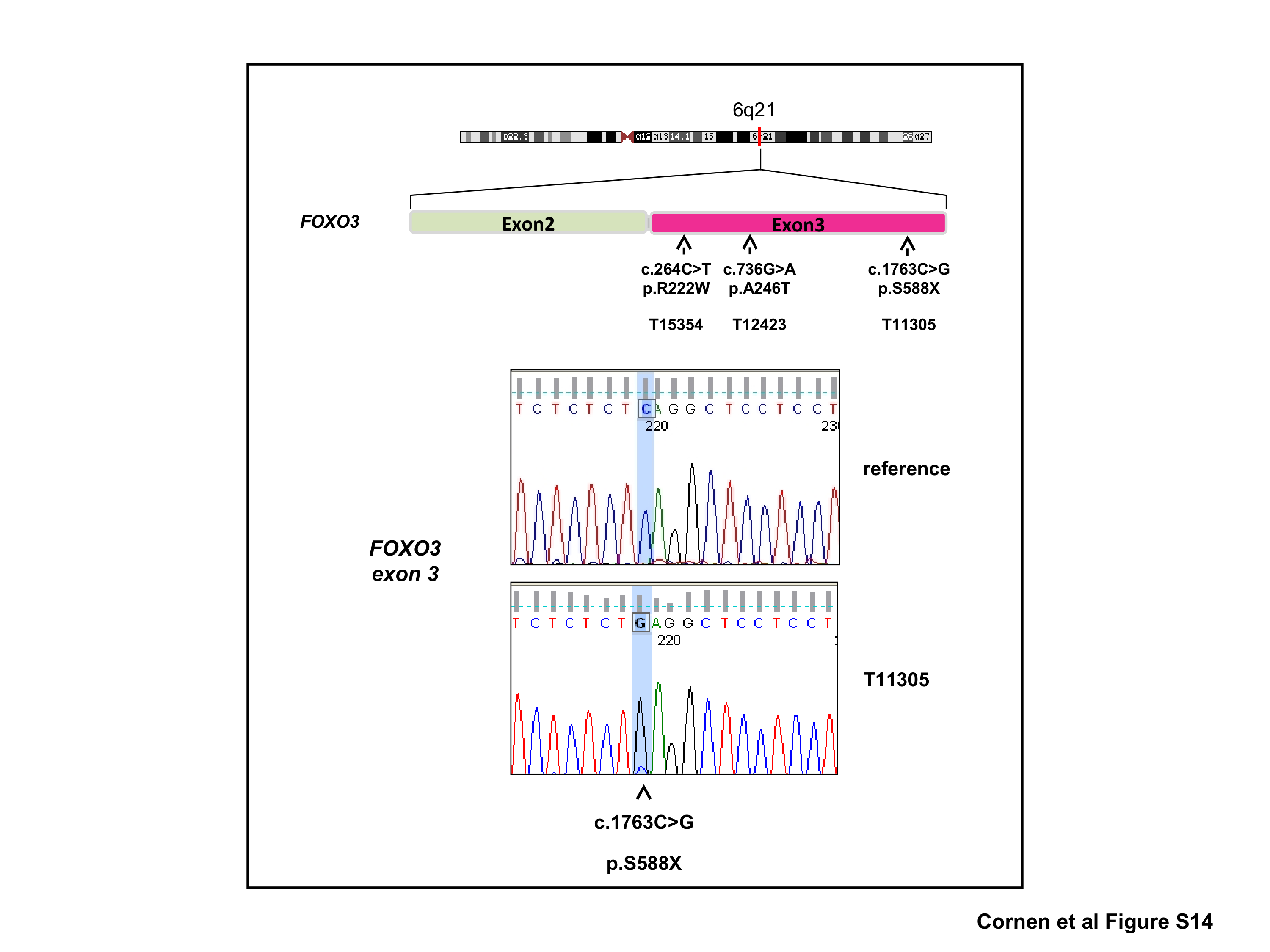

Supplement: Figure S14 — Examples of mutation of FOXO3 in breast cancer. From top to the bottom, in the 6q21 chromosomal band, the FOXO3 gene spans the chr6:108,881,026–109,005,971 region. Exons 2 and 3 of FOXO3 gene were analyzed for mutations. Sequence profile of the mutated FOXO3 allele, demonstrating base change in the forward sequence at the position indicated by an arrow. The corresponding sequence is shown above. (TIF) [file pone.0081843.s014.tif]

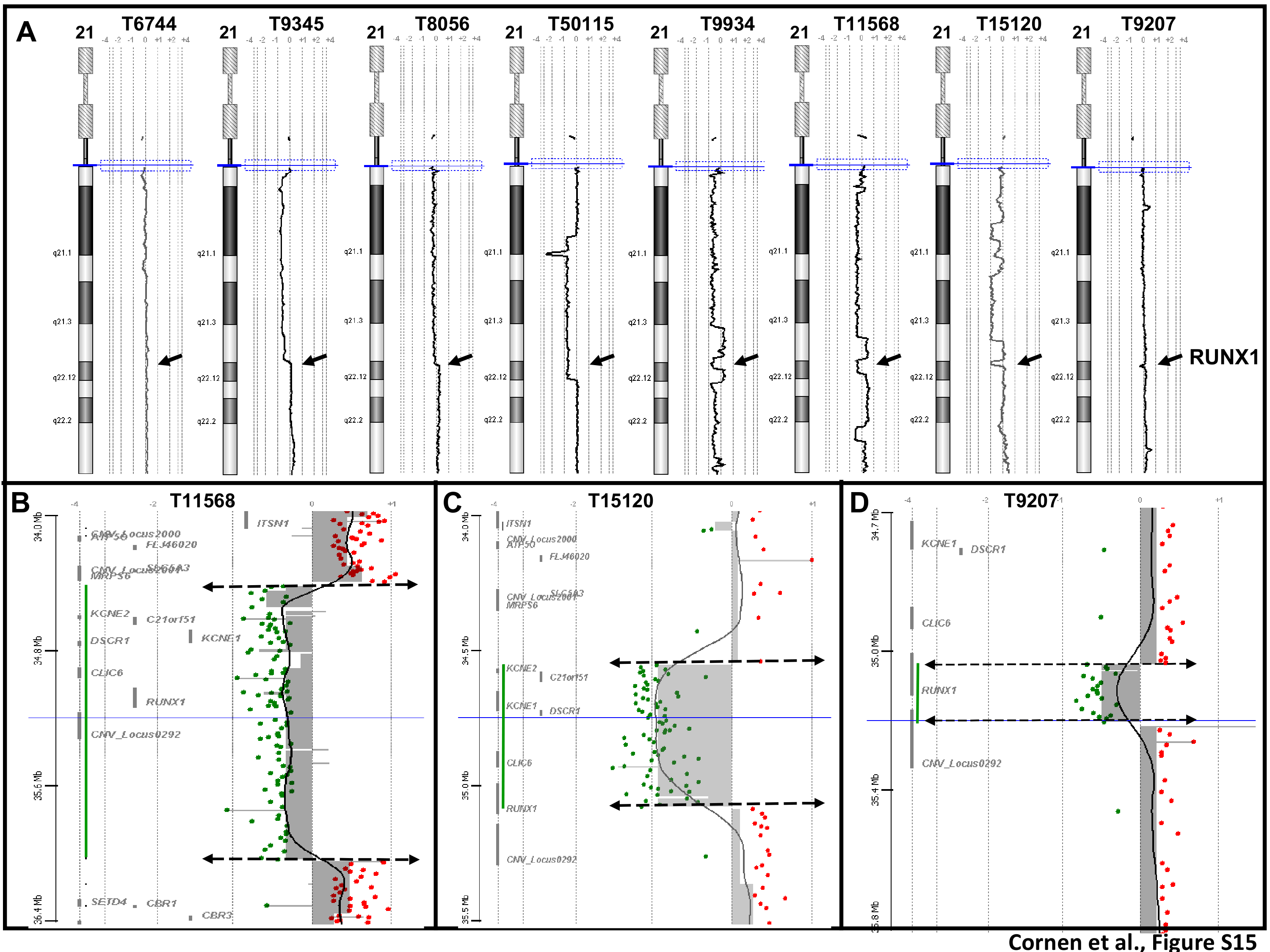

Supplement: Figure S15 — Examples of aCGH profiles showing RUNX1 losses. (A) From left to right, aCGH profiles of chromosome 21 in cases T6744 (no apparent CNA), T9345, T8056, T50115, T9934, T11568, T15120, and T9207. Arrow shows RUNX1 location on each genomic profile. Results show that RUNX1 is targeted by potential breaks in T8056, T9207 and T15120, but also by regional deletions (samples T50115, T9934, T11568, T15120, and T9207). The genomic profiles of T11568 (B), T15120 (C), and T9207 (D) BCs presenting the smallest regional deletions were established with CGH analytics® software (Agilent Technologies), from centromere to telomere, within the genomic intervals [34.0–36.4 Mb] of the long arm of the chromosome 21. The common smallest deleted region observed in T9207 (D) involves RUNX1. (TIF) [file pone.0081843.s015.tif]
