# Supplementary material for: Candidate Luminal B Breast Cancer Genes Identified by Genome, Gene Expression and DNA Methylation Profiling
Source: PLoS One. 2014 Jan 9;9(1):e81843. doi: 10.1371/journal.pone.0081843 (PMC3886975; doi:10.1371/journal.pone.0081843)
Supplement: Results and References S1 — In this section, results about: validation of our methylation approach; DNA methylation level associated with the other breast cancer molecular subtypes; and specific deregulated gene expression in relation with the DNA methylation level variation associated with the other breast cancer molecular subtypes; are presented with supplementary references. (DOCX) [file pone.0081843.s016.docx]

**Results and references S1.**

**SI/ Results.**

***Note:*** *References with numbers are included in the main text, the others are in SI_supplementary references.*

**Validation of our methylation approach.** To validate our approach on promoter array and our calculation method, we compared the DNA methylation of *RASSF1* gene promoter in 48 tumor samples quantified by using two independent technologies, i.e. promoter array (Agilent Technologies) and EpiTyper^TM^ Mass-ARRAY^®^ system (SEQUENOM^®^, USA). For the detection and quantitative analysis of DNA methylation, the EpiTyper approach used eight amplicons spanning the region chr3:50,349,000-50,352,780, which includes the *RASSF1* promoter and was covered by nine *RASSF1* oligonucleotides on the promoter array (**Fig. S10B**). Based on the methylation variation on the 95 CpG present within this region and measured by EpiTyper, the hierarchical clustering distinguished ER+ and ER- tumors (Fisher, p=4.3x10^-3^) (**Fig. S11A**). For each sample, median methylation values (EpiTyper) were calculated, with four informative amplicons overlapping oligoprobes (see arrows on **Fig. S11A**) used to calculate a methylation score. We observed a strong correlation between median methylation values and methylation score (Pearson correlation=0.66, p=4.6x10^-7^) calculated from data established by EpiTyper and promoter array approaches, respectively (**Fig. S11B**).

**DNA methylation associated with the other breast cancer molecular subtypes.** In luminal A tumors, 318 and 289 promoters had a DNA methylation level higher and lower than in the other subtypes, respectively. Among them, 108 and 64 had a DNA methylation level higher and lower than in NB tissues (t test, FDR<0.05). In ERBB2 tumors, 77 and 105 promoters had a DNA methylation level higher and lower than in the other subtypes, respectively. Among them, 39 and 50 had a DNA methylation level higher and lower than in NB tissues (t test, FDR<0.05). In basal tumors, 1388 and 1518 promoters had a DNA methylation level higher and lower than in the other subtypes, respectively. Among them, 519 and 506 had a DNA methylation level higher and lower than in NB tissues (t test, FDR<0.05).

**The other molecular subtypes and specific deregulated gene expression in relation with the DNA methylation level variation.** In the luminal A tumors, among the 172 promoters associated with a significant DNA methylation variation (compared to the other subtypes and NB tissues), 13 corresponding genes presented a deregulated expression (9 and 4 were down and upregulated, respectively) (correlation<-0.40) (**Table S4E**). 23% (3 genes) were found significantly deregulated in relation with their level of DNA methylation in TCGA data, (Table S4E). Among them, high DNA methylation level targeted promoters of *CSTA*, *HIVEP2* and *MARKS* genes previously reported as TSGs or associated with tumor progression (**Table S4E**). Only higher *DEGS2* gene expression was more significantly observed in luminal A compared to the other subtypes (t test, p<0.05) (**Fig. S12B**).

In the ERBB2 tumors, among the 89 promoters associated with a significant DNA methylation variation, 7 corresponding genes presented a deregulated expression (3 and 4 were down and upregulated, respectively) (correlation<-0.40) (**Table S4E**). 14% (1 gene) was found significantly deregulated in relation with their level of DNA methylation in TCGA data, (**Table S4E**).

Only lower *EZH1* and higher *C17ORF37, CRKRS, PERLD1* gene expression were more significantly observed in ERBB2 cases compared to the other subtypes (t test, p<0.05) (**Fig. S12C**). The lower *EZH1* mRNA observed in ERBB2 tumors could perturb methylation of histone H3 lys27 (H3K27) and function in the maintenance of embryonic stem cell pluripotency and plasticity.

In the basal tumors, among the 1025 promoters associated with a significant DNA methylation variation, 102 corresponding genes presented a deregulated expression (57 and 45 were down and upregulated, respectively) (correlation<-0.40) (**Table S4E**). 61% (62 gene) was found significantly deregulated in relation with their level of DNA methylation in TCGA data, (**Table S4E**). Among them, high DNA methylation level targeted promoters of *DUSP4*, *DUSP5*, *HRASLS3*, *NAT1*, *PDCD4*, *PTPN13* and *RHOB* genes previously reported as TSGs or associated with tumor progression (**Table S4E**). Only lower *CMBL, CRAT, CXXC5, DUSP4, ERGIC1, FOXA1, FZD4, LOC400451, NOSTRIN, RHOB, SCNN1A, TBC1D9, TFF3, WFS1* and higher *CDC2L6, CDKN2A, ELF5, EN1, FABP7, FOXC1, FOXM1, HJURP, HPDL, IMPA2, KIAA1804, MCM5, MEX3A, MGC23985, PTPLA, TEM38A, ST8SIA1* gene expression were more significantly observed in basal compared to the other subtypes (t test, p<0.05) (**Fig. 4D and S12D1-D4**).

We thus identified 7, 1, 4 and 31 genes that presented a deregulated gene expression in relation with DNA methylation variation of their promoter specifically in luminal B, luminal A, ERRB2 and basal molecular subtypes, respectively.

**Subtype-specific candidates presenting gene expression deregulation in relation with CNA and with DNA methylation aberrations.** In the luminal A tumors, none gene was deregulated in relation with CNAs and with methylation level (**Table S4F**).

In the ERBB2 tumors, no gene was downregulated in relation with copy number loss and with high DNA methylation of the promoters (**Table S4F**). However, the overexpression of *C17ORF37, CRKRS, PERLD1* present within the *ERBB2* amplicon [17] could result from the amplification or from a concomitant significant lower DNA methylation level of their promoters (**Table S4F** and **Fig. S12C**).

In the basal tumors, 17 genes were downregulated in relation with copy number loss and with the high methylation level of the promoter (**Table S4F**). However, only *CRAT, CXXC5, DUSP4, ERGIC1, FOXA1, LOC400451, TBC1D9*, and *WFS1* genes were the most significantly downregulated in basal tumors (**Table S4F** and **Fig. S12D1-D4**). Conversely, 12 genes were upregulated in relation with copy number gain or amplification and/or with the low DNA methylation level of the promoter (**Table S4F**). However, only *CDC2L6, FABP7, FOXC1, FOXM1* and *PTPLA* genes were the most significantly upregulated in basal tumors (**Table S4F** and **Fig. S12D1-D4**).

**SI/Supplementary references**

Afonja O, Juste D, Das S, Matsuhashi S, Samuels HH (2004) [Induction of PDCD4 tumor suppressor gene expression by RAR agonists, antiestrogen and HER-2/neu antagonist in breast cancer cells. Evidence for a role in apoptosis.](http://www.ncbi.nlm.nih.gov/pubmed/15361828) Oncogene 23: 8135-8145.

Arai E, Chiku S, Mori T, Gotoh M, Nakagawa T, et al. (2012) [Single-CpG-resolution methylome analysis identifies clinicopathologically aggressive CpG island methylator phenotype clear cell renal cell carcinomas.](http://www.ncbi.nlm.nih.gov/pubmed/22610075) Carcinogenesis 33: 1487-1493.

Armes JE, Hammet F, de Silva M, Ciciulla J, Ramus SJ, et al. (2004) [Candidate tumor-suppressor genes on chromosome arm 8p in early-onset and high-grade breast cancers.](http://www.ncbi.nlm.nih.gov/pubmed/15184884) Oncogene 23: 5697-5702.

Barry WT, Kernagis DN, Dressman HK, Griffis RJ, Hunter JD, et al. (2010) [Intratumor heterogeneity and precision of microarray-based predictors of breast cancer biology and clinical outcome.](http://www.ncbi.nlm.nih.gov/pubmed/20368555) J Clin Oncol 28: 2198-2206.

[Birnbaum DJ](http://www.ncbi.nlm.nih.gov/pubmed?term=Birnbaum%20DJ%5BAuthor%5D&cauthor=true&cauthor_uid=21412932), [Adélaïde J](http://www.ncbi.nlm.nih.gov/pubmed?term=Ad%C3%A9la%C3%AFde%20J%5BAuthor%5D&cauthor=true&cauthor_uid=21412932), [Mamessier E](http://www.ncbi.nlm.nih.gov/pubmed?term=Mamessier%20E%5BAuthor%5D&cauthor=true&cauthor_uid=21412932), [Finetti P](http://www.ncbi.nlm.nih.gov/pubmed?term=Finetti%20P%5BAuthor%5D&cauthor=true&cauthor_uid=21412932), [Lagarde A](http://www.ncbi.nlm.nih.gov/pubmed?term=Lagarde%20A%5BAuthor%5D&cauthor=true&cauthor_uid=21412932), et al. (2011) Genome profiling of pancreatic adenocarcinoma. [Genes Chromosomes Cancer](http://www.ncbi.nlm.nih.gov/pubmed/21412932) 50: 456-465.

Bonnefoi H, Potti A, Delorenzi M, Mauriac L, Campone M, et al. (2007) [Validation of gene signatures that predict the response of breast cancer to neoadjuvant chemotherapy: a substudy of the EORTC 10994/BIG 00-01 clinical trial.](http://www.ncbi.nlm.nih.gov/pubmed/18024211) Lancet Oncol 8: 1071-1078.

Bos PD, Zhang XH, Nadal C, Shu W, Gomis RR, et al. (2009) [Genes that mediate breast cancer metastasis to the brain.](http://www.ncbi.nlm.nih.gov/pubmed/19421193) Nature 459: 1005-1009.

[Brockschmidt A](http://www.ncbi.nlm.nih.gov/pubmed?term=Brockschmidt%20A%5BAuthor%5D&cauthor=true&cauthor_uid=22427331), [Trost D](http://www.ncbi.nlm.nih.gov/pubmed?term=Trost%20D%5BAuthor%5D&cauthor=true&cauthor_uid=22427331), [Peterziel H](http://www.ncbi.nlm.nih.gov/pubmed?term=Peterziel%20H%5BAuthor%5D&cauthor=true&cauthor_uid=22427331), [Zimmermann K](http://www.ncbi.nlm.nih.gov/pubmed?term=Zimmermann%20K%5BAuthor%5D&cauthor=true&cauthor_uid=22427331), [Ehrler M](http://www.ncbi.nlm.nih.gov/pubmed?term=Ehrler%20M%5BAuthor%5D&cauthor=true&cauthor_uid=22427331), et al. (2012) KIAA1797/FOCAD encodes a novel focal adhesion protein with tumour suppressor function in gliomas. [Brain](http://www.ncbi.nlm.nih.gov/pubmed?term=FOCAD) 2012; 135: 1027-1041.

Charboneau AL, Singh V, Yu T, Newsham IF (2002) [Suppression of growth and increased cellular attachment after expression of DAL-1 in MCF-7 breast cancer cells.](http://www.ncbi.nlm.nih.gov/pubmed/12115567) Int J Cancer 100: 181-188.

Chen DT, Nasir A, Culhane A, Venkataramu C, Fulp W, et al. (2010) [Proliferative genes dominate malignancy-risk gene signature in histologically-normal breast tissue.](http://www.ncbi.nlm.nih.gov/pubmed/19266279) Breast Cancer Res Treat 119: 335-346.

[Chou JL](http://www.ncbi.nlm.nih.gov/pubmed?term=Chou%20JL%5BAuthor%5D&cauthor=true&cauthor_uid=20065949), [Su HY](http://www.ncbi.nlm.nih.gov/pubmed?term=Su%20HY%5BAuthor%5D&cauthor=true&cauthor_uid=20065949), [Chen LY](http://www.ncbi.nlm.nih.gov/pubmed?term=Chen%20LY%5BAuthor%5D&cauthor=true&cauthor_uid=20065949), [Liao YP](http://www.ncbi.nlm.nih.gov/pubmed?term=Liao%20YP%5BAuthor%5D&cauthor=true&cauthor_uid=20065949), [Hartman-Frey C](http://www.ncbi.nlm.nih.gov/pubmed?term=Hartman-Frey%20C%5BAuthor%5D&cauthor=true&cauthor_uid=20065949), et al. (2010) Promoter hypermethylation of FBXO32, a novel TGF-beta/SMAD4 target gene and tumor suppressor, is associated with poor prognosis in human ovarian cancer. [Lab Invest](http://www.ncbi.nlm.nih.gov/pubmed/20065949) 90: 414-425.

[Christopher SA](http://www.ncbi.nlm.nih.gov/pubmed?term=Christopher%20SA%5BAuthor%5D&cauthor=true&cauthor_uid=12438261), [Diegelman P](http://www.ncbi.nlm.nih.gov/pubmed?term=Diegelman%20P%5BAuthor%5D&cauthor=true&cauthor_uid=12438261), [Porter CW](http://www.ncbi.nlm.nih.gov/pubmed?term=Porter%20CW%5BAuthor%5D&cauthor=true&cauthor_uid=12438261), [Kruger WD](http://www.ncbi.nlm.nih.gov/pubmed?term=Kruger%20WD%5BAuthor%5D&cauthor=true&cauthor_uid=12438261) (2002) Methylthioadenosine phosphorylase, a gene frequently codeleted with p16(cdkN2a/ARF), acts as a tumor suppressor in a breast cancer cell line. [Cancer Res](http://www.ncbi.nlm.nih.gov/pubmed?term=Christopher%20AND%202002%20AND%20CDKN2A) 62: 6639-6644.

Couderc B, Pradines A, Rafii A, Golzio M, Deviers A, et al. (2008) [In vivo restoration of RhoB expression leads to ovarian tumor regression.](http://www.ncbi.nlm.nih.gov/pubmed/18340357) Cancer Gene Ther 15:456-464.

Dedeurwaerder S, Desmedt C, Calonne E, Singhal SK, Haibe-Kains B, et al. (2011) [DNA methylation profiling reveals a predominant immune component in breast cancers.](http://www.ncbi.nlm.nih.gov/pubmed/21910250) EMBO Mol Med 3: 726-741.

Delage B, Fennell DA, Nicholson L, McNeish I, Lemoine NR, et al. (2010) [Arginine deprivation and argininosuccinate synthetase expression in the treatment of cancer.](http://www.ncbi.nlm.nih.gov/pubmed/20104527) Int J Cancer 126: 2762-2772.

[Desmedt C](http://www.ncbi.nlm.nih.gov/pubmed?term=Desmedt%20C%5BAuthor%5D&cauthor=true&cauthor_uid=17545524), [Piette F](http://www.ncbi.nlm.nih.gov/pubmed?term=Piette%20F%5BAuthor%5D&cauthor=true&cauthor_uid=17545524), [Loi S](http://www.ncbi.nlm.nih.gov/pubmed?term=Loi%20S%5BAuthor%5D&cauthor=true&cauthor_uid=17545524), [Wang Y](http://www.ncbi.nlm.nih.gov/pubmed?term=Wang%20Y%5BAuthor%5D&cauthor=true&cauthor_uid=17545524), [Lallemand F](http://www.ncbi.nlm.nih.gov/pubmed?term=Lallemand%20F%5BAuthor%5D&cauthor=true&cauthor_uid=17545524), et al. (2007) Strong time dependence of the 76-gene prognostic signature for node-negative breast cancer patients in the TRANSBIG multicenter independent validation series. Clin Cancer Res 13: 3207-3214.

[Desmedt C](http://www.ncbi.nlm.nih.gov/pubmed?term=Desmedt%20C%5BAuthor%5D&cauthor=true&cauthor_uid=21422418), [Di Leo A](http://www.ncbi.nlm.nih.gov/pubmed?term=Di%20Leo%20A%5BAuthor%5D&cauthor=true&cauthor_uid=21422418), [de Azambuja E](http://www.ncbi.nlm.nih.gov/pubmed?term=de%20Azambuja%20E%5BAuthor%5D&cauthor=true&cauthor_uid=21422418), [Larsimont D](http://www.ncbi.nlm.nih.gov/pubmed?term=Larsimont%20D%5BAuthor%5D&cauthor=true&cauthor_uid=21422418), [Haibe-Kains B](http://www.ncbi.nlm.nih.gov/pubmed?term=Haibe-Kains%20B%5BAuthor%5D&cauthor=true&cauthor_uid=21422418), et al. (2011) Multifactorial approach to predicting resistance to anthracyclines. [J Clin Oncol](http://www.ncbi.nlm.nih.gov/pubmed/?term=Desmedt++AND+2011+and+breast+cancer+and+J+Clin+Oncol+journal) 29: 1578-1586.

Expression Project for Oncology (expO) (2005): <https://expo.intgen.org/geo>.

[Farmer P](http://www.ncbi.nlm.nih.gov/pubmed?term=Farmer%20P%5BAuthor%5D&cauthor=true&cauthor_uid=15897907), [Bonnefoi H](http://www.ncbi.nlm.nih.gov/pubmed?term=Bonnefoi%20H%5BAuthor%5D&cauthor=true&cauthor_uid=15897907), [Becette V](http://www.ncbi.nlm.nih.gov/pubmed?term=Becette%20V%5BAuthor%5D&cauthor=true&cauthor_uid=15897907), [Tubiana-Hulin M](http://www.ncbi.nlm.nih.gov/pubmed?term=Tubiana-Hulin%20M%5BAuthor%5D&cauthor=true&cauthor_uid=15897907), [Fumoleau P](http://www.ncbi.nlm.nih.gov/pubmed?term=Fumoleau%20P%5BAuthor%5D&cauthor=true&cauthor_uid=15897907), et al. (2005) Identification of molecular apocrine breast tumours by microarray analysis. Oncogene 24: 4660-4671.

Fournier G, Cabaud O, Josselin E, Chaix A, Adélaïde J, et al. (2011) Loss of AF6/Afadin, a marker of poor outcome in breast cancer, induces cell migration, invasiveness and tumor growth. Oncogene 30:3862-3874.

Fujii H, Gabrielson E, Takagaki T, Ohtsuji M, Ohtsuji N, Hino O (2005) [Frequent down-regulation of HIVEP2 in human breast cancer.](http://www.ncbi.nlm.nih.gov/pubmed/15868437) Breast Cancer Res Treat 91: 103-112.

Gelsi-Boyer V, Trouplin V, Adélaïde J, Aceto N, Remy V, et al. (2008) Genome profiling of chronic myelomonocytic leukemia: frequent alterations of RAS and RUNX1 genes. BMC Cancer 8: 299.

Gelsi-Boyer V, Trouplin V, Adélaïde J, Bonansea J, Cervera N, et al. (2009) [Mutations of polycomb-associated gene ASXL1 in myelodysplastic syndromes and chronic myelomonocytic leukaemia.](http://www.ncbi.nlm.nih.gov/pubmed/19388938?ordinalpos=2&itool=EntrezSystem2.PEntrez.Pubmed.Pubmed_ResultsPanel.Pubmed_DefaultReportPanel.Pubmed_RVDocSum) Br J Haematol 145: 788-800.

Glondu-Lassis M, Dromard M, Lacroix-Triki M, Nirdé P, Puech C, et al. (2010) [PTPL1/PTPN13 regulates breast cancer cell aggressiveness through direct inactivation of Src kinase.](http://www.ncbi.nlm.nih.gov/pubmed/20501847) Cancer Res 70: 5116-5126.

[Guedj M](http://www.ncbi.nlm.nih.gov/pubmed?term=Guedj%20M%5BAuthor%5D&cauthor=true&cauthor_uid=21785460), [Marisa L](http://www.ncbi.nlm.nih.gov/pubmed?term=Marisa%20L%5BAuthor%5D&cauthor=true&cauthor_uid=21785460), [de Reynies A](http://www.ncbi.nlm.nih.gov/pubmed?term=de%20Reynies%20A%5BAuthor%5D&cauthor=true&cauthor_uid=21785460), [Orsetti B](http://www.ncbi.nlm.nih.gov/pubmed?term=Orsetti%20B%5BAuthor%5D&cauthor=true&cauthor_uid=21785460), [Schiappa R](http://www.ncbi.nlm.nih.gov/pubmed?term=Schiappa%20R%5BAuthor%5D&cauthor=true&cauthor_uid=21785460), et al. (2012) A refined molecular taxonomy of breast cancer. [Oncogene](http://www.ncbi.nlm.nih.gov/pubmed/?term=Guedj++AND+2011+and+breast+cancer+and+oncogene) 31: 1196-1206.

Hatzis C, Pusztai L, Valero V, Booser DJ, Esserman L, et al. (2011) A genomic predictor of response and survival following taxane-anthracycline chemotherapy for invasive breast cancer. JAMA 305:1873-81.

[Herschkowitz JI](http://www.ncbi.nlm.nih.gov/pubmed?term=Herschkowitz%20JI%5BAuthor%5D&cauthor=true&cauthor_uid=17493263), [Simin K](http://www.ncbi.nlm.nih.gov/pubmed?term=Simin%20K%5BAuthor%5D&cauthor=true&cauthor_uid=17493263), [Weigman VJ](http://www.ncbi.nlm.nih.gov/pubmed?term=Weigman%20VJ%5BAuthor%5D&cauthor=true&cauthor_uid=17493263), [Mikaelian I](http://www.ncbi.nlm.nih.gov/pubmed?term=Mikaelian%20I%5BAuthor%5D&cauthor=true&cauthor_uid=17493263), [Usary J](http://www.ncbi.nlm.nih.gov/pubmed?term=Usary%20J%5BAuthor%5D&cauthor=true&cauthor_uid=17493263), et al. (2007) Identification of conserved gene expression features between murine mammary carcinoma models and human breast tumors. Genome Biol 8: R76.

Hess KR, Anderson K, Symmans WF, Valero V, Ibrahim N, et al. (2006) Pharmacogenomic predictor of sensitivity to preoperative chemotherapy with paclitaxel and fluorouracil, doxorubicin, and cyclophosphamide in breast cancer. J Clin Oncol 24: 4236-4244.

Hicks J, Krasnitz A, Lakshmi B, Navin NE, Riggs M, et al. (2006) Novel patterns of genome rearrangement and their association with survival in breast cancer. Genome Res 16: 1465-1479.

[Hoadley KA](http://www.ncbi.nlm.nih.gov/pubmed?term=Hoadley%20KA%5BAuthor%5D&cauthor=true&cauthor_uid=17663798), [Weigman VJ](http://www.ncbi.nlm.nih.gov/pubmed?term=Weigman%20VJ%5BAuthor%5D&cauthor=true&cauthor_uid=17663798), [Fan C](http://www.ncbi.nlm.nih.gov/pubmed?term=Fan%20C%5BAuthor%5D&cauthor=true&cauthor_uid=17663798), [Sawyer LR](http://www.ncbi.nlm.nih.gov/pubmed?term=Sawyer%20LR%5BAuthor%5D&cauthor=true&cauthor_uid=17663798), [He X](http://www.ncbi.nlm.nih.gov/pubmed?term=He%20X%5BAuthor%5D&cauthor=true&cauthor_uid=17663798), et al. (2007) EGFR associated expression profiles vary with breast tumor subtype. BMC Genomics 8:258.

Hodson DJ, Janas ML, Galloway A, Bell SE, Andrews S, et al. (2010) [Deletion of the RNA-binding proteins ZFP36L1 and ZFP36L2 leads to perturbed thymic development and T lymphoblastic leukemia.](http://www.ncbi.nlm.nih.gov/pubmed/20622884) Nat Immunol 11: 717-724.

Hoeflich KP, O'Brien C, Boyd Z, Cavet G, Guerrero S, et al. (2009) [In vivo antitumor activity of MEK and phosphatidylinositol 3-kinase inhibitors in basal-like breast cancer models.](http://www.ncbi.nlm.nih.gov/pubmed/19567590) Clin Cancer Res 15: 4649-4664.

Honma K, Tsuzuki S, Nakagawa M, Tagawa H, Nakamura S, et al. (2009) [TNFAIP3/A20 functions as a novel tumor suppressor gene in several subtypes of non-Hodgkin lymphomas.](http://www.ncbi.nlm.nih.gov/pubmed/19608751) Blood 114: 2467-2475.

[Hu N](http://www.ncbi.nlm.nih.gov/pubmed?term=Hu%20N%5BAuthor%5D&cauthor=true&cauthor_uid=17134496), [Wang C](http://www.ncbi.nlm.nih.gov/pubmed?term=Wang%20C%5BAuthor%5D&cauthor=true&cauthor_uid=17134496), [Hu Y](http://www.ncbi.nlm.nih.gov/pubmed?term=Hu%20Y%5BAuthor%5D&cauthor=true&cauthor_uid=17134496), [Yang HH](http://www.ncbi.nlm.nih.gov/pubmed?term=Yang%20HH%5BAuthor%5D&cauthor=true&cauthor_uid=17134496), [Kong LH](http://www.ncbi.nlm.nih.gov/pubmed?term=Kong%20LH%5BAuthor%5D&cauthor=true&cauthor_uid=17134496), et al. (2006) Genome-wide loss of heterozygosity and copy number alteration in esophageal squamous cell carcinoma using the Affymetrix GeneChip Mapping 10 K array. BMC Genomics 7: 299.

Hu Z, Fan C, Livasy C, He X, Oh DS, et al. (2009) A compact VEGF signature associated with distant metastases and poor outcomes. BMC Med 7: 9.

Huang KT, Takano EA, Mikeska T, Byrne DJ, Dobrovic A, Fox SB (2011) [Aberrant DNA methylation but not mutation of CITED4 is associated with alteration of HIF-regulated genes in breast cancer.](http://www.ncbi.nlm.nih.gov/pubmed/21755341) Breast Cancer Res Treat 130: 319-329.

Ivshina AV, George J, Senko O, Mow B, Putti TC, et al. (2006) [Genetic reclassification of histologic grade delineates new clinical subtypes of breast cancer.](http://www.ncbi.nlm.nih.gov/pubmed/17079448) Cancer Res 66:10292-10301.

[Iwamoto T](http://www.ncbi.nlm.nih.gov/pubmed?term=Iwamoto%20T%5BAuthor%5D&cauthor=true&cauthor_uid=21191116), [Bianchini G](http://www.ncbi.nlm.nih.gov/pubmed?term=Bianchini%20G%5BAuthor%5D&cauthor=true&cauthor_uid=21191116), [Booser D](http://www.ncbi.nlm.nih.gov/pubmed?term=Booser%20D%5BAuthor%5D&cauthor=true&cauthor_uid=21191116), [Qi Y](http://www.ncbi.nlm.nih.gov/pubmed?term=Qi%20Y%5BAuthor%5D&cauthor=true&cauthor_uid=21191116), [Coutant C](http://www.ncbi.nlm.nih.gov/pubmed?term=Coutant%20C%5BAuthor%5D&cauthor=true&cauthor_uid=21191116), et al. (2011) Gene pathways associated with prognosis and chemotherapy sensitivity in molecular subtypes of breast cancer. J Natl Cancer Inst 103: 264-272.

Iwanaga E, Nanri T, Mitsuya H, Asou N (2011) [Mutation in the RNA binding protein TIS11D/ZFP36L2 is associated with the pathogenesis of acute leukemia.](http://www.ncbi.nlm.nih.gov/pubmed/21109922) Int J Oncol 38: 25-31.

Jovanovic J, Rønneberg JA, Tost J, Kristensen V (2010) [The epigenetics of breast cancer.](http://www.ncbi.nlm.nih.gov/pubmed/20627830) Mol Oncol 4: 242-254.

[Karube K](http://www.ncbi.nlm.nih.gov/pubmed?term=Karube%20K%5BAuthor%5D&cauthor=true&cauthor_uid=21690554), [Nakagawa M](http://www.ncbi.nlm.nih.gov/pubmed?term=Nakagawa%20M%5BAuthor%5D&cauthor=true&cauthor_uid=21690554), [Tsuzuki S](http://www.ncbi.nlm.nih.gov/pubmed?term=Tsuzuki%20S%5BAuthor%5D&cauthor=true&cauthor_uid=21690554), [Takeuchi I](http://www.ncbi.nlm.nih.gov/pubmed?term=Takeuchi%20I%5BAuthor%5D&cauthor=true&cauthor_uid=21690554), [Honma K](http://www.ncbi.nlm.nih.gov/pubmed?term=Honma%20K%5BAuthor%5D&cauthor=true&cauthor_uid=21690554), et al. (2011) Identification of FOXO3 and PRDM1 as tumor-suppressor gene candidates in NK-cell neoplasms by genomic and functional analyses. [Blood](http://www.ncbi.nlm.nih.gov/pubmed/21690554##) 118: 3195-3204.

[Klein A](http://www.ncbi.nlm.nih.gov/pubmed?term=Klein%20A%5BAuthor%5D&cauthor=true&cauthor_uid=17410534), [Wessel R](http://www.ncbi.nlm.nih.gov/pubmed?term=Wessel%20R%5BAuthor%5D&cauthor=true&cauthor_uid=17410534), [Graessmann M](http://www.ncbi.nlm.nih.gov/pubmed?term=Graessmann%20M%5BAuthor%5D&cauthor=true&cauthor_uid=17410534), [Jürgens M](http://www.ncbi.nlm.nih.gov/pubmed?term=J%C3%BCrgens%20M%5BAuthor%5D&cauthor=true&cauthor_uid=17410534), [Petersen I](http://www.ncbi.nlm.nih.gov/pubmed?term=Petersen%20I%5BAuthor%5D&cauthor=true&cauthor_uid=17410534), ,et al. (2007) Comparison of gene expression data from human and mouse breast cancers: identification of a conserved breast tumor gene set. Int J Cancer 121: 683-688.

Korde LA, Lusa L, McShane L, Lebowitz PF, Lukes L, et al. (2010) [Gene expression pathway analysis to predict response to neoadjuvant docetaxel and capecitabine for breast cancer.](http://www.ncbi.nlm.nih.gov/pubmed/20012355) Breast Cancer Res Treat 119: 685-699.

Küçük C, Iqbal J, Hu X, Gaulard P, De Leval L, et al. (2011) [PRDM1 is a tumor suppressor gene in natural killer cell malignancies.](http://www.ncbi.nlm.nih.gov/pubmed/22143801) Proc Natl Acad Sci U S A; 108: 20119-20124.

[Lee S](http://www.ncbi.nlm.nih.gov/pubmed?term=Lee%20S%5BAuthor%5D&cauthor=true&cauthor_uid=22751464), [Stewart S](http://www.ncbi.nlm.nih.gov/pubmed?term=Stewart%20S%5BAuthor%5D&cauthor=true&cauthor_uid=22751464), [Nagtegaal I](http://www.ncbi.nlm.nih.gov/pubmed?term=Nagtegaal%20I%5BAuthor%5D&cauthor=true&cauthor_uid=22751464), [Luo J](http://www.ncbi.nlm.nih.gov/pubmed?term=Luo%20J%5BAuthor%5D&cauthor=true&cauthor_uid=22751464), [Wu Y](http://www.ncbi.nlm.nih.gov/pubmed?term=Wu%20Y%5BAuthor%5D&cauthor=true&cauthor_uid=22751464), et al. (2012) Differentially expressed genes regulating the progression of ductal carcinoma in situ to invasive breast cancer. [Cancer Res](http://www.ncbi.nlm.nih.gov/pubmed/22751464) 72: 4574-4586.

Letessier A, Garrido-Urbani S, Ginestier C, Esterni B, Monville F, et al. (2007) Correlated break at PARK2/FRA6E and loss of AF-6/Afadin protein expression are associated with poor outcome in breast cancer. Oncogene 2007; 26: 298-307.

Li CF, MacDonald JR, Wei RY, Ray J, Lau K, et al. (2007) [Human sterile alpha motif domain 9, a novel gene identified as down-regulated in aggressive fibromatosis, is absent in the mouse.](http://www.ncbi.nlm.nih.gov/pubmed/17407603) BMC Genomics 8: 92.

Luo D, Huang H, Lu ML, Zhao GF, Chang J, et al. (2012a) [Abnormal expression of adhesion protein Bves is associated with gastric cancer progression and poor survival.](http://www.ncbi.nlm.nih.gov/pubmed/22109561) Pathol Oncol Res 18: 491-497.

Luo D, Lu ML, Zhao GF, Huang H, Zheng MY, et al. (2012b) [Reduced Popdc3 expression correlates with high risk and poor survival in patients with gastric cancer.](http://www.ncbi.nlm.nih.gov/pubmed/22654436) World J Gastroenterol 18: 2423-2429.

Marty B, Maire V, Gravier E, Rigaill G, Vincent-Salomon A, et al. (2008) [Frequent PTEN genomic alterations and activated phosphatidylinositol 3-kinase pathway in basal-like breast cancer cells.](http://www.ncbi.nlm.nih.gov/pubmed/19055754) Breast Cancer Res 10: R101.

Merritt WM, Lin YG, Han LY, Kamat AA, Spannuth WA, et al. (2008) Dicer, Drosha, and outcomes in patients with ovarian cancer. N Engl J Med 359:2641-2650.

Michel S, Kloor M, Singh S, Gdynia G, Roth W, von Knebel et al. (2010) [Coding microsatellite instability analysis in microsatellite unstable small intestinal adenocarcinomas identifies MARCKS as a common target of inactivation.](http://www.ncbi.nlm.nih.gov/pubmed/19852062) Mol Carcinog 49: 175-182.

Miller LD, Smeds J, George J, [Vega VB](http://www.ncbi.nlm.nih.gov/pubmed?term=Vega%20VB%5BAuthor%5D&cauthor=true&cauthor_uid=16141321), [Vergara L](http://www.ncbi.nlm.nih.gov/pubmed?term=Vergara%20L%5BAuthor%5D&cauthor=true&cauthor_uid=16141321), et al. (2005) An expression signature for p53 status in human breast cancer predicts mutation status, transcriptional effects, and patient survival. Proc Natl Acad Sci U S A 102: 13550-13555.

Miller WR, Larionov A (2010) Changes in expression of oestrogen regulated and proliferation genes with neoadjuvant treatment highlight heterogeneity of clinical resistance to the aromatase inhibitor, letrozole. Breast Cancer Res 12: R52.

Millour J., Constantinidou D., Stavropoulou A.V., Wilson M.S., Myatt S.S., et al. (2010) FOXM1 is a transcriptional target of ERalpha and has a critical role in breast cancer endocrine sensitivity and resistance. Oncogene 29: 2983–2995.

Minn AJ, Gupta GP, Siegel PM, Bos PD, Shu W, et al. (2005) Genes that mediate breast cancer metastasis to lung. Nature 436: 518-524.

Negorev DG, Vladimirova OV, Kossenkov AV, Nikonova EV, Demarest RM, et al. (2010) [Sp100 as a potent tumor suppressor: accelerated senescence and rapid malignant transformation of human fibroblasts through modulation of an embryonic stem cell program.](http://www.ncbi.nlm.nih.gov/pubmed/21118961) Cancer Res 70: 9991-10001.

Nik-Zainal S, Alexandrov LB, Wedge DC, Van Loo P, Greenman CD, et al. (2012) [Mutational processes molding the genomes of 21 breast cancers.](http://www.ncbi.nlm.nih.gov/pubmed/22608084) Cell 149: 979-993.

[Novellino L](http://www.ncbi.nlm.nih.gov.gate2.inist.fr/pubmed?term=Novellino%20L%5BAuthor%5D&cauthor=true&cauthor_uid=18276111), [De Filippo A](http://www.ncbi.nlm.nih.gov.gate2.inist.fr/pubmed?term=De%20Filippo%20A%5BAuthor%5D&cauthor=true&cauthor_uid=18276111), [Deho P](http://www.ncbi.nlm.nih.gov.gate2.inist.fr/pubmed?term=Deho%20P%5BAuthor%5D&cauthor=true&cauthor_uid=18276111), [Perrone F](http://www.ncbi.nlm.nih.gov.gate2.inist.fr/pubmed?term=Perrone%20F%5BAuthor%5D&cauthor=true&cauthor_uid=18276111), [Pilotti S](http://www.ncbi.nlm.nih.gov.gate2.inist.fr/pubmed?term=Pilotti%20S%5BAuthor%5D&cauthor=true&cauthor_uid=18276111), et al. (2008) PTPRK negatively regulates transcriptional activity of wild type and mutated oncogenic beta-catenin and affects membrane distribution of beta-catenin/E-cadherin complexes in cancer cells. [Cell Signal](http://www.ncbi.nlm.nih.gov.gate2.inist.fr/pubmed/18276111) 20: 872-883.

[Oh JL](http://www.ncbi.nlm.nih.gov/pubmed?term=Oh%20JL%5BAuthor%5D&cauthor=true&cauthor_uid=17075114), [Dryden MJ](http://www.ncbi.nlm.nih.gov/pubmed?term=Dryden%20MJ%5BAuthor%5D&cauthor=true&cauthor_uid=17075114), [Woodward WA](http://www.ncbi.nlm.nih.gov/pubmed?term=Woodward%20WA%5BAuthor%5D&cauthor=true&cauthor_uid=17075114), [Yu TK](http://www.ncbi.nlm.nih.gov/pubmed?term=Yu%20TK%5BAuthor%5D&cauthor=true&cauthor_uid=17075114), [Tereffe W](http://www.ncbi.nlm.nih.gov/pubmed?term=Tereffe%20W%5BAuthor%5D&cauthor=true&cauthor_uid=17075114), et al. (2006) Locoregional control of clinically diagnosed multifocal or multicentric breast cancer after neoadjuvant chemotherapy and locoregional therapy. J Clin Oncol 24: 4971-4975.

Oliveira AM, Ross JS, Fletcher JA (2005) [Tumor suppressor genes in breast cancer: the gatekeepers and the caretakers.](http://www.ncbi.nlm.nih.gov/pubmed/16468415) Am J Clin Pathol 124 Suppl: S16-28.

Park SY, Kwon HJ, Choi Y, Lee HE, Kim SW, et al. (2012) [Distinct patterns of promoter CpG island methylation of breast cancer subtypes are associated with stem cell phenotypes.](http://www.ncbi.nlm.nih.gov/pubmed/22037257) Mod Pathol 25: 185-196.

Parker JS, Mullins M, Cheang MC, Leung S, Voduc D, et al. (2009) [Supervised risk predictor of breast cancer based on intrinsic subtypes.](http://www.ncbi.nlm.nih.gov/pubmed/19204204) J Clin Oncol 27:1160-1167.

[Parker JS](http://www.ncbi.nlm.nih.gov/pubmed?term=Parker%20JS%5BAuthor%5D&cauthor=true&cauthor_uid=19204204), [Mullins M](http://www.ncbi.nlm.nih.gov/pubmed?term=Mullins%20M%5BAuthor%5D&cauthor=true&cauthor_uid=19204204), [Cheang MC](http://www.ncbi.nlm.nih.gov/pubmed?term=Cheang%20MC%5BAuthor%5D&cauthor=true&cauthor_uid=19204204), [Leung S](http://www.ncbi.nlm.nih.gov/pubmed?term=Leung%20S%5BAuthor%5D&cauthor=true&cauthor_uid=19204204), [Voduc D](http://www.ncbi.nlm.nih.gov/pubmed?term=Voduc%20D%5BAuthor%5D&cauthor=true&cauthor_uid=19204204), et al. (2009) Supervised risk predictor of breast cancer based on intrinsic subtypes. J Clin Oncol 27: 1160-1167.

Peck AR, Witkiewicz AK, Liu C, Klimowicz AC, Stringer GA, et al. (2012) [Low levels of Stat5a protein in breast cancer are associated with tumor progression and unfavorable clinical outcomes.](http://www.ncbi.nlm.nih.gov/pubmed/23036105) Breast Cancer Res 14: R130.

Perreard L, Fan C, Quackenbush JF, Mullins M, Gauthier NP, et al. (2006) Classification and risk stratification of invasive breast carcinomas using a real-time quantitative RT-PCR assay. Breast Cancer Res 8: R23.

Piwko W, Olma MH, Held M, Bianco JN, Pedrioli PG, Hofmann K, et al. [RNAi-based screening identifies the Mms22L-Nfkbil2 complex as a novel regulator of DNA replication in human cells.](http://www.ncbi.nlm.nih.gov/pubmed/21113133) EMBO J 2010; 29: 4210-4222.

[Popovici V](http://www.ncbi.nlm.nih.gov/pubmed?term=Popovici%20V%5BAuthor%5D&cauthor=true&cauthor_uid=20064235), [Chen W](http://www.ncbi.nlm.nih.gov/pubmed?term=Chen%20W%5BAuthor%5D&cauthor=true&cauthor_uid=20064235), [Gallas BG](http://www.ncbi.nlm.nih.gov/pubmed?term=Gallas%20BG%5BAuthor%5D&cauthor=true&cauthor_uid=20064235), [Hatzis C](http://www.ncbi.nlm.nih.gov/pubmed?term=Hatzis%20C%5BAuthor%5D&cauthor=true&cauthor_uid=20064235), [Shi W](http://www.ncbi.nlm.nih.gov/pubmed?term=Shi%20W%5BAuthor%5D&cauthor=true&cauthor_uid=20064235), [Samuelson FW](http://www.ncbi.nlm.nih.gov/pubmed?term=Samuelson%20FW%5BAuthor%5D&cauthor=true&cauthor_uid=20064235), et al. (2010) Effect of training-sample size and classification difficulty on the accuracy of genomic predictors. Breast Cancer Res 12: R5.

[Prat A](http://www.ncbi.nlm.nih.gov/pubmed?term=Prat%20A%5BAuthor%5D&cauthor=true&cauthor_uid=20813035), [Parker JS](http://www.ncbi.nlm.nih.gov/pubmed?term=Parker%20JS%5BAuthor%5D&cauthor=true&cauthor_uid=20813035), [Karginova O](http://www.ncbi.nlm.nih.gov/pubmed?term=Karginova%20O%5BAuthor%5D&cauthor=true&cauthor_uid=20813035), [Fan C](http://www.ncbi.nlm.nih.gov/pubmed?term=Fan%20C%5BAuthor%5D&cauthor=true&cauthor_uid=20813035), [Livasy C](http://www.ncbi.nlm.nih.gov/pubmed?term=Livasy%20C%5BAuthor%5D&cauthor=true&cauthor_uid=20813035), et al. (2010) Phenotypic and molecular characterization of the claudin-low intrinsic subtype of breast cancer. Breast Cancer Res 12: R68.

[Schmidt M](http://www.ncbi.nlm.nih.gov/pubmed?term=Schmidt%20M%5BAuthor%5D&cauthor=true&cauthor_uid=18593943), [Böhm D](http://www.ncbi.nlm.nih.gov/pubmed?term=B%C3%B6hm%20D%5BAuthor%5D&cauthor=true&cauthor_uid=18593943), [von Törne C](http://www.ncbi.nlm.nih.gov/pubmed?term=von%20T%C3%B6rne%20C%5BAuthor%5D&cauthor=true&cauthor_uid=18593943), [Steiner E](http://www.ncbi.nlm.nih.gov/pubmed?term=Steiner%20E%5BAuthor%5D&cauthor=true&cauthor_uid=18593943), [Puhl A](http://www.ncbi.nlm.nih.gov/pubmed?term=Puhl%20A%5BAuthor%5D&cauthor=true&cauthor_uid=18593943), et al. (2008) The humoral immune system has a key prognostic impact in node-negative breast cancer. Cancer Res 68: 5405-5413.

Shen X, Liu Y, Hsu YJ, Fujiwara Y, Kim J, et al. (2008) [EZH1 mediates methylation on histone H3 lysine 27 and complements EZH2 in maintaining stem cell identity and executing pluripotency.](http://www.ncbi.nlm.nih.gov/pubmed/19026780) Mol Cell 32: 491-502.

[Silver DP](http://www.ncbi.nlm.nih.gov/pubmed?term=Silver%20DP%5BAuthor%5D&cauthor=true&cauthor_uid=20100965), [Richardson AL](http://www.ncbi.nlm.nih.gov/pubmed?term=Richardson%20AL%5BAuthor%5D&cauthor=true&cauthor_uid=20100965), [Eklund AC](http://www.ncbi.nlm.nih.gov/pubmed?term=Eklund%20AC%5BAuthor%5D&cauthor=true&cauthor_uid=20100965), [Wang ZC](http://www.ncbi.nlm.nih.gov/pubmed?term=Wang%20ZC%5BAuthor%5D&cauthor=true&cauthor_uid=20100965), [Szallasi Z](http://www.ncbi.nlm.nih.gov/pubmed?term=Szallasi%20Z%5BAuthor%5D&cauthor=true&cauthor_uid=20100965), et al. (20010) Efficacy of neoadjuvant Cisplatin in triple-negative breast cancer. [J Clin Oncol](http://www.ncbi.nlm.nih.gov/pubmed/?term=Silver+AND+2010+and+breast+cancer+and+J+Clin+Oncol+journal) 28:1145-1153.

[Sotiriou C](http://www.ncbi.nlm.nih.gov/pubmed?term=Sotiriou%20C%5BAuthor%5D&cauthor=true&cauthor_uid=16478745), [Wirapati P](http://www.ncbi.nlm.nih.gov/pubmed?term=Wirapati%20P%5BAuthor%5D&cauthor=true&cauthor_uid=16478745), [Loi S](http://www.ncbi.nlm.nih.gov/pubmed?term=Loi%20S%5BAuthor%5D&cauthor=true&cauthor_uid=16478745), [Harris A](http://www.ncbi.nlm.nih.gov/pubmed?term=Harris%20A%5BAuthor%5D&cauthor=true&cauthor_uid=16478745), [Fox S](http://www.ncbi.nlm.nih.gov/pubmed?term=Fox%20S%5BAuthor%5D&cauthor=true&cauthor_uid=16478745), et al. (2006) Gene expression profiling in breast cancer: understanding the molecular basis of histologic grade to improve prognosis. J Natl Cancer Inst 98: 262-272.

Sunami E, Shinozaki M, Sim MS, Nguyen SL, Vu AT, et al. (2008) [Estrogen receptor and HER2/neu status affect epigenetic differences of tumor-related genes in primary breast tumors.](http://www.ncbi.nlm.nih.gov/pubmed/18485221) Breast Cancer Res 10:R46.

[Tabchy A](http://www.ncbi.nlm.nih.gov/pubmed?term=Tabchy%20A%5BAuthor%5D&cauthor=true&cauthor_uid=20829329), [Valero V](http://www.ncbi.nlm.nih.gov/pubmed?term=Valero%20V%5BAuthor%5D&cauthor=true&cauthor_uid=20829329), [Vidaurre T](http://www.ncbi.nlm.nih.gov/pubmed?term=Vidaurre%20T%5BAuthor%5D&cauthor=true&cauthor_uid=20829329), [Lluch A](http://www.ncbi.nlm.nih.gov/pubmed?term=Lluch%20A%5BAuthor%5D&cauthor=true&cauthor_uid=20829329), [Gomez H](http://www.ncbi.nlm.nih.gov/pubmed?term=Gomez%20H%5BAuthor%5D&cauthor=true&cauthor_uid=20829329), et al. (2010) Evaluation of a 30-gene paclitaxel, fluorouracil, doxorubicin, and cyclophosphamide chemotherapy response predictor in a multicenter randomized trial in breast cancer. Clin Cancer Res 16: 5351-5361.

Tan J, Yang X, Zhuang L, Jiang X, Chen W, et al. (2007) [Pharmacologic disruption of Polycomb-repressive complex 2-mediated gene repression selectively induces apoptosis in cancer cells.](http://www.ncbi.nlm.nih.gov/pubmed/17437993) Genes Dev 21:1050-1063.

Tiang JM, Butcher NJ, Cullinane C, Humbert PO, Minchin RF (2011) [RNAi-mediated knock-down of arylamine N-acetyltransferase-1 expression induces E-cadherin up-regulation and cell-cell contact growth inhibition.](http://www.ncbi.nlm.nih.gov/pubmed/21347396) PLoS One 6: e17031.

[Ueda K](http://www.ncbi.nlm.nih.gov/pubmed?term=Ueda%20K%5BAuthor%5D&cauthor=true&cauthor_uid=12944906), [Arakawa H](http://www.ncbi.nlm.nih.gov/pubmed?term=Arakawa%20H%5BAuthor%5D&cauthor=true&cauthor_uid=12944906), [Nakamura Y](http://www.ncbi.nlm.nih.gov/pubmed?term=Nakamura%20Y%5BAuthor%5D&cauthor=true&cauthor_uid=12944906) (2003) Dual-specificity phosphatase 5 (DUSP5) as a direct transcriptional target of tumor suppressor p53. [Oncogene](http://www.ncbi.nlm.nih.gov/pubmed/?term=Ueda+AND+2003+AND+DUSP5) 22: 5586-5591.

Ursini-Siegel J, Rajput AB, Lu H, Sanguin-Gendreau V, Zuo D, et al. (2007) [Elevated expression of DecR1 impairs ErbB2/Neu-induced mammary tumor development.](http://www.ncbi.nlm.nih.gov/pubmed/17636013) Mol Cell Biol 27: 6361-6371.

van de Vijver MJ, He YD, van't Veer LJ, Dai H, Hart AA, et al. (2002) [A gene-expression signature as a predictor of survival in breast cancer.](http://www.ncbi.nlm.nih.gov/pubmed/12490681) N Engl J Med 347: 1999-2009.

van 't Veer LJ, Dai H, van de Vijver MJ, He YD, Hart AA, Mao M, et al. (2002) [Gene expression profiling predicts clinical outcome of breast cancer.](http://www.ncbi.nlm.nih.gov/pubmed/11823860) Nature 415: 530-536.

[Wang Y](http://www.ncbi.nlm.nih.gov/pubmed?term=Wang%20Y%5BAuthor%5D&cauthor=true&cauthor_uid=15721472), [Klijn JG](http://www.ncbi.nlm.nih.gov/pubmed?term=Klijn%20JG%5BAuthor%5D&cauthor=true&cauthor_uid=15721472), [Zhang Y](http://www.ncbi.nlm.nih.gov/pubmed?term=Zhang%20Y%5BAuthor%5D&cauthor=true&cauthor_uid=15721472), [Sieuwerts AM](http://www.ncbi.nlm.nih.gov/pubmed?term=Sieuwerts%20AM%5BAuthor%5D&cauthor=true&cauthor_uid=15721472), [Look MP](http://www.ncbi.nlm.nih.gov/pubmed?term=Look%20MP%5BAuthor%5D&cauthor=true&cauthor_uid=15721472), [Yang F](http://www.ncbi.nlm.nih.gov/pubmed?term=Yang%20F%5BAuthor%5D&cauthor=true&cauthor_uid=15721472), et al. (2005) Gene-expression profiles to predict distant metastasis of lymph-node-negative primary breast cancer. Lancet 365: 671-679.

[Weigelt B](http://www.ncbi.nlm.nih.gov/pubmed?term=Weigelt%20B%5BAuthor%5D&cauthor=true&cauthor_uid=16230372), [Hu Z](http://www.ncbi.nlm.nih.gov/pubmed?term=Hu%20Z%5BAuthor%5D&cauthor=true&cauthor_uid=16230372), [He X](http://www.ncbi.nlm.nih.gov/pubmed?term=He%20X%5BAuthor%5D&cauthor=true&cauthor_uid=16230372), [Livasy C](http://www.ncbi.nlm.nih.gov/pubmed?term=Livasy%20C%5BAuthor%5D&cauthor=true&cauthor_uid=16230372), [Carey LA](http://www.ncbi.nlm.nih.gov/pubmed?term=Carey%20LA%5BAuthor%5D&cauthor=true&cauthor_uid=16230372), et al. (2005) Molecular portraits and 70-gene prognosis signature are preserved throughout the metastatic process of breast cancer. Cancer Res 65: 9155-9158.

[Yanatatsaneejit P](http://www.ncbi.nlm.nih.gov/pubmed?term=Yanatatsaneejit%20P%5BAuthor%5D&cauthor=true&cauthor_uid=17689134), [Chalermchai T](http://www.ncbi.nlm.nih.gov/pubmed?term=Chalermchai%20T%5BAuthor%5D&cauthor=true&cauthor_uid=17689134), [Kerekhanjanarong V](http://www.ncbi.nlm.nih.gov/pubmed?term=Kerekhanjanarong%20V%5BAuthor%5D&cauthor=true&cauthor_uid=17689134), [Shotelersuk K](http://www.ncbi.nlm.nih.gov/pubmed?term=Shotelersuk%20K%5BAuthor%5D&cauthor=true&cauthor_uid=17689134), [Supiyaphun P](http://www.ncbi.nlm.nih.gov/pubmed?term=Supiyaphun%20P%5BAuthor%5D&cauthor=true&cauthor_uid=17689134), et al. (2008) Promoter hypermethylation of CCNA1, RARRES1, and HRASLS3 in nasopharyngeal carcinoma. [Oral Oncol](http://www.ncbi.nlm.nih.gov/pubmed?term=HRASLS3%20loss%20and%20cancer) 44:400-406.

Yao J, Weremowicz S, Feng B, Gentleman RC, Marks JR, et al. (2006) [Combined cDNA array comparative genomic hybridization and serial analysis of gene expression analysis of breast tumor progression.](http://www.ncbi.nlm.nih.gov/pubmed/16618726) Cancer Res 66: 4065-4078.

Yeh SH, Wu DC, Tsai CY, Kuo TJ, Yu WC, et al. (2006) [Genetic characterization of fas-associated phosphatase-1 as a putative tumor suppressor gene on chromosome 4q21.3 in hepatocellular carcinoma.](http://www.ncbi.nlm.nih.gov/pubmed/16489062) Clin Cancer Res 12: 1097-1108.

[Yu K](http://www.ncbi.nlm.nih.gov/pubmed?term=Yu%20K%5BAuthor%5D&cauthor=true&cauthor_uid=18636107), [Ganesan K](http://www.ncbi.nlm.nih.gov/pubmed?term=Ganesan%20K%5BAuthor%5D&cauthor=true&cauthor_uid=18636107), [Tan LK](http://www.ncbi.nlm.nih.gov/pubmed?term=Tan%20LK%5BAuthor%5D&cauthor=true&cauthor_uid=18636107), [Laban M](http://www.ncbi.nlm.nih.gov/pubmed?term=Laban%20M%5BAuthor%5D&cauthor=true&cauthor_uid=18636107), [Wu J](http://www.ncbi.nlm.nih.gov/pubmed?term=Wu%20J%5BAuthor%5D&cauthor=true&cauthor_uid=18636107), et al. (2008) A precisely regulated gene expression cassette potently modulates metastasis and survival in multiple solid cancers. PLoS Genet 4: e1000129.

Zhang L, Anglesio MS, O'Sullivan M, Zhang F, Yang G, et al. (2007) [The E3 ligase HACE1 is a critical chromosome 6q21 tumor suppressor involved in multiple cancers.](http://www.ncbi.nlm.nih.gov/pubmed/17694067) Nat Med 13: 1060-9.

[Zhang Y](http://www.ncbi.nlm.nih.gov/pubmed?term=Zhang%20Y%5BAuthor%5D&cauthor=true&cauthor_uid=18821012), [Sieuwerts AM](http://www.ncbi.nlm.nih.gov/pubmed?term=Sieuwerts%20AM%5BAuthor%5D&cauthor=true&cauthor_uid=18821012), [McGreevy M](http://www.ncbi.nlm.nih.gov/pubmed?term=McGreevy%20M%5BAuthor%5D&cauthor=true&cauthor_uid=18821012), [Casey G](http://www.ncbi.nlm.nih.gov/pubmed?term=Casey%20G%5BAuthor%5D&cauthor=true&cauthor_uid=18821012), et al. (2009) The 76-gene signature defines high-risk patients that benefit from adjuvant tamoxifen therapy. Breast Cancer Res Treat 116:303-309.

[Zhou D](http://www.ncbi.nlm.nih.gov.gate2.inist.fr/pubmed?term=Zhou%20D%5BAuthor%5D&cauthor=true&cauthor_uid=17068076), [Ye JJ](http://www.ncbi.nlm.nih.gov.gate2.inist.fr/pubmed?term=Ye%20JJ%5BAuthor%5D&cauthor=true&cauthor_uid=17068076), [Li Y](http://www.ncbi.nlm.nih.gov.gate2.inist.fr/pubmed?term=Li%20Y%5BAuthor%5D&cauthor=true&cauthor_uid=17068076), [Lui K](http://www.ncbi.nlm.nih.gov.gate2.inist.fr/pubmed?term=Lui%20K%5BAuthor%5D&cauthor=true&cauthor_uid=17068076), [Chen S](http://www.ncbi.nlm.nih.gov.gate2.inist.fr/pubmed?term=Chen%20S%5BAuthor%5D&cauthor=true&cauthor_uid=17068076) (2006) The molecular basis of the interaction between the proline-rich SH3-binding motif of PNRC and estrogen receptor alpha. [Nucleic Acids Res](http://www.ncbi.nlm.nih.gov.gate2.inist.fr/pubmed?term=Dujin%20Zhou%202006%20PNRC) 34: 5974-5986.
